# Elicitation of Neutralizing Antibodies Targeting the V2 Apex of the HIV Envelope Trimer in a Wild-Type Animal Model

**DOI:** 10.1016/j.celrep.2017.10.089

**Published:** 2018-01-28

**Authors:** James E. Voss, Raiees Andrabi, Laura E. McCoy, Natalia de Val, Roberta P. Fuller, Terrence Messmer, Ching-Yao Su, Devin Sok, Salar N. Khan, Fernando Garces, Laura K. Pritchard, Richard T. Wyatt, Andrew B. Ward, Max Crispin, Ian A. Wilson, Dennis R. Burton

(Cell Reports *21*, 222–235; October 3, 2017)

In the originally published version of this article, a statement in the discussion was not updated to reflect the data finally presented in Figure 1. The original publication contained the following statement: (3) “Differing V2-apex iP reactivity. The most effective immunogens, C108 and CRF250, bind all of the PG9, CH01, and CAP256 prototype iPs, while WITO and MGRM8 are neutralized by only two of these antibodies. Further, WITO and MGRM8 SOSIPs showed the poorest binding to iP/UCA antibodies by BLI, suggesting there are potentially fewer ways for them to trigger naive BCRs.” This statement has now been replaced by the following revision: (3) “Differing V2-apex iP reactivity. One of the more effective immunogens, CRF250, binds all of the PG9, CH01, and CAP256 precursor antibodies, suggesting there are potentially multiple ways for this trimer specifically to engage V2-apex-directed naive BCRs and giving it a potential advantage with regard to nAb elicitation.”

The authors apologize for this error.

