# Supplementary material for: Elicitation of Neutralizing Antibodies Targeting the V2 Apex of the HIV Envelope Trimer in a Wild-Type Animal Model
Source: Cell Rep. 2017 Oct 3;21(1):222–35. doi: 10.1016/j.celrep.2017.09.024 (PMC5640805; doi:10.1016/j.celrep.2017.09.024)
Supplement: Document S1. Figures S1–S5 [file mmc1.pdf]

Cell Reports, Volume *21*

Supplemental Information

Elicitation of Neutralizing Antibodies

Targeting the V2 Apex of the HIV Envelope

Trimer in a Wild-Type Animal Model

James E. Voss, Raiees Andrabi, Laura E. McCoy, Natalia de Val, Roberta P. Fuller, Terrence Messmer, Ching-Yao Su, Devin Sok, Salar N. Khan, Fernando Garces, Laura K. Pritchard, Richard T. Wyatt, Andrew B. Ward, Max Crispin, Ian A. Wilson, and Dennis R. Burton

A

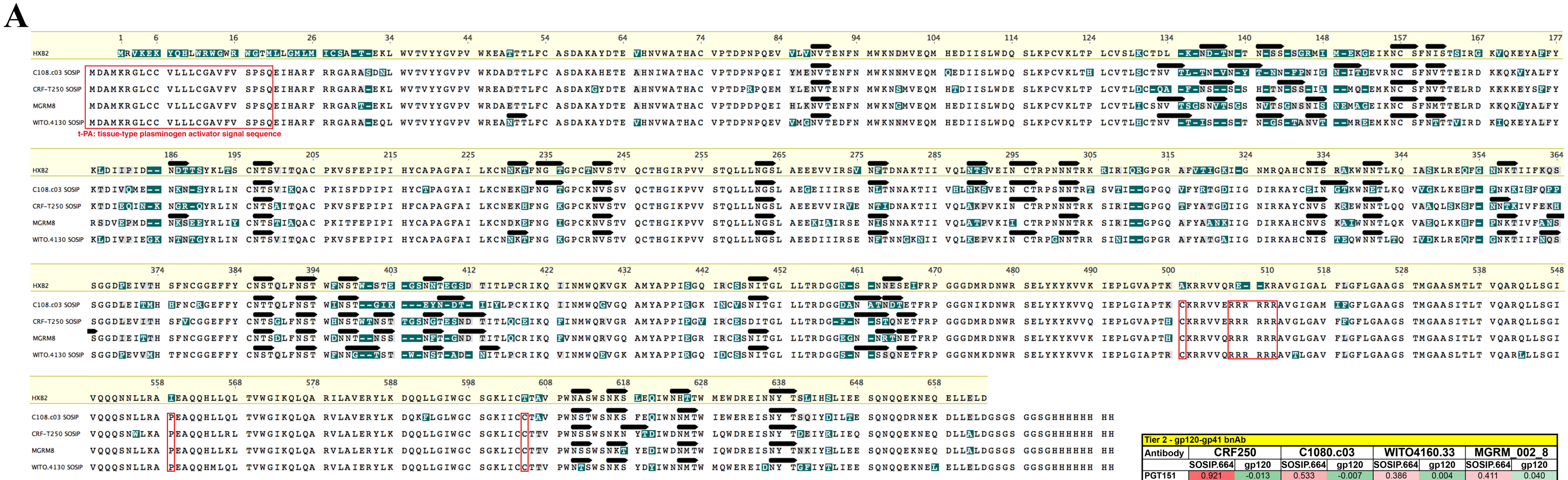

B

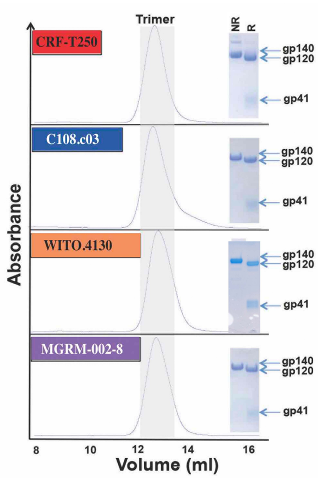

C

Tier 2 - V2 Apex bnAb

Antibody	CRF250		C1080.c03		WITO4160.33		MGRM_002_8	
	SOSIP.664	gp120	SOSIP.664	gp120	SOSIP.664	gp120	SOSIP.664	gp120
PG9	0.605	0.047	0.480	0.175	0.428	0.067	0.218	0.173
PG16	0.639	0.039	0.531	0.108	0.360	0.055	0.287	0.113
PGT141	0.664	0.021	0.368	0.002	0.680	0.020	0.487	0.057
PGT142	0.787	0.022	0.369	-0.010	0.791	0.008	0.515	0.053
PGT143	0.710	0.022	0.385	-0.002	0.773	0.000	0.343	0.059
PGT144	0.581	0.025	0.192	-0.005	0.667	0.005	0.070	0.078
PGT145	0.878	-0.002	0.614	0.001	0.834	0.014	0.469	0.034
PGDM1400	0.817	-0.007	0.798	0.003	0.598	0.012	0.452	0.052
CH01	0.324	-0.013	0.454	0.008	0.255	0.014	0.146	0.079
CH02	0.232	-0.014	0.301	-0.008	0.291	0.006	0.091	0.039
CH03	0.347	-0.005	0.401	-0.004	0.233	0.002	0.099	0.062
CH04	0.244	-0.013	0.351	0.001	0.188	0.008	0.123	0.079
CAP256.01	0.634	0.041	0.160	0.030	0.059	0.061	0.268	0.076
CAP256.02	0.779	0.018	0.206	0.008	0.089	0.034	0.399	0.066
CAP256.03	0.821	0.031	0.383	0.038	0.065	0.052	0.475	0.063
CAP256.04	0.746	0.031	0.282	0.036	0.071	0.049	0.413	0.063
CAP256.05	0.856	0.025	0.161	0.027	0.067	0.034	0.346	0.061
CAP256.06	0.507	0.014	0.037	0.004	0.663	0.016	0.247	0.047
CAP256.07	0.476	-0.003	0.025	-0.002	0.043	0.010	0.177	0.039
CAP256.08	0.676	0.014	0.430	0.029	0.048	0.040	0.431	0.097
CAP256.09	0.725	0.015	0.416	0.038	0.044	0.037	0.493	0.073
CAP256.10	0.566	0.018	0.165	0.028	0.096	0.040	0.329	0.054
CAP256.11	0.627	0.039	0.082	0.036	0.075	0.043	0.323	0.076
CAP256.12	0.348	0.022	0.021	0.032	0.054	0.034	0.202	0.072
CAP256.I1	0.509	0.042	0.022	0.031	0.055	0.062	0.178	0.075
CAP256.I2	0.751	0.028	0.120	0.030	0.058	0.054	0.338	0.065
**non-neutralizing V2 Apex**								
2909	0.066	0.037	0.032	0.039	0.063	0.053	0.062	0.071
C108G	0.078	0.054	0.055	0.068	0.078	0.077	0.069	0.083
830A	0.052	0.020	0.052	0.206	0.177	0.482	0.135	0.135
697	0.069	0.052	0.035	0.233	0.092	0.182	0.109	0.245

Tier 2 - V3-glycan bnAb

Antibody	CRF250		C1080.c03		WITO4160.33		MGRM_002_8	
	SOSIP.664	gp120	SOSIP.664	gp120	SOSIP.664	gp120	SOSIP.664	gp120
PGT121	0.813	0.166	0.036	0.002	0.316	0.164	0.351	0.073
PGT122	0.691	0.063	0.021	0.015	0.169	0.118	0.236	0.224
PGT123	0.899	0.173	0.037	0.001	0.378	0.215	0.425	0.245
PGT124	0.762	0.240	0.007	0.003	0.236	0.308	0.381	0.431
PGT125	0.651	0.157	0.530	0.197	0.653	0.065	0.486	0.481
PGT126	0.676	0.185	0.545	0.106	0.610	0.081	0.539	0.635
PGT127	0.508	0.048	0.277	-0.003	0.216	0.016	0.335	0.405
PGT128	0.731	0.190	0.478	0.111	0.622	0.052	0.464	0.651
PGT130	0.595	0.156	0.431	0.133	0.361	0.047	0.314	0.542
PGT131	0.661	0.144	0.421	0.084	0.207	0.068	0.275	0.514
PGT133	0.804	0.232	0.024	0.022	0.227	0.325	0.382	-0.001
10-1074	0.823	0.270	0.033	0.021	0.267	0.362	0.415	0.431
PGT135	0.510	0.116	0.006	-0.066	0.304	0.247	0.193	0.119
PGT136	0.067	0.022	0.012	-0.004	0.328	0.199	0.143	0.079
PGT137	0.539	0.131	0.012	-0.016	0.467	0.168	0.193	0.202
2G12	0.559	0.241	0.314	-0.010	0.556	0.229	0.420	0.215

Tier 2 - CD4 binding site bnAb

Antibody	CRF250		C1080.c03		WITO4160.33		MGRM_002_8	
	SOSIP.664	gp120	SOSIP.664	gp120	SOSIP.664	gp120	SOSIP.664	gp120
VRC01	0.063	0.011	0.050	0.130	0.143	0.114	0.140	0.275
VRC03	0.062	0.010	0.017	0.023	0.253	0.038	0.077	0.104
VRC06	0.023	-0.003	-0.004	0.011	0.037	0.022	0.037	0.064
HJ16	0.044	0.005	0.022	0.023	0.065	0.050	0.058	0.065
12A12	0.113	0.046	0.138	0.231	0.175	0.198	0.166	0.375
NIH45-46	0.409	0.024	0.056	0.112	0.134	0.061	0.188	0.354
PGV04	0.049	0.033	0.012	0.093	0.097	0.088	0.094	0.271
CH103	0.075	0.060	0.029	0.047	0.074	0.076	0.096	0.299
3BNC60	0.108	0.015	0.083	0.144	0.289	0.142	0.204	0.281
3BNC117	0.090	0.009	0.061	0.135	0.290	0.092	0.170	0.253
b6	0.128	0.614	0.041	0.062	0.145	0.354	0.282	0.651
b12	0.057	-0.025	0.281	0.266	0.251	0.299	0.401	0.542
F105	0.037	0.452	-0.019	-0.019	0.075	0.412	0.164	0.514
CD4-IgG2	0.382	0.589	0.382	0.589	0.293	0.565	0.540	0.850

Tier 2 - gp120-gp41 bnAb

Antibody	CRF250		C1080.c03		WITO4160.33		MGRM_002_8	
	SOSIP.664	gp120	SOSIP.664	gp120	SOSIP.664	gp120	SOSIP.664	gp120
PGT151	0.921	-0.013	0.533	-0.007	0.386	0.004	0.411	0.040
PGT152	0.846	-0.013	0.615	-0.002	0.328	0.001	0.316	0.041
PGT153	0.616	-0.004	0.294	0.012	0.094	0.020	0.088	0.035
PGT154	0.774	0.009	0.192	0.021	0.090	0.040	0.188	0.049
PGT155	0.854	0.022	0.458	0.026	0.244	0.033	0.297	0.058
PGT156	0.865	0.024	0.512	0.034	0.241	0.045	0.369	0.085
PGT157	0.905	0.051	0.384	0.044	0.213	0.078	0.307	0.091
PGT158	0.928	0.033	0.485	0.033	0.203	0.048	0.310	0.078
8ANC195	-0.002	-0.023	0.056	0.152	0.163	-0.016	0.214	-0.001
*35022	0.373	-0.058	0.079	-0.066	0.023	-0.063	0.122	-0.011

Non-neutralizing V3

Antibody	CRF250		C1080.c03		WITO4160.33		MGRM_002_8	
	SOSIP.664	gp120	SOSIP.664	gp120	SOSIP.664	gp120	SOSIP.664	gp120
447-52D	0.207	0.402	0.042	0.028	0.585	0.591	0.413	0.405
2557	0.356	0.608	0.112	0.157	0.367	0.321	0.354	0.640
4022	0.385	0.637	0.085	0.161	0.323	0.331	0.391	0.639
3074	0.290	0.682	0.242	0.497	0.317	0.467	0.362	0.693
3791	0.333	0.733	0.124	0.275	0.074	0.065	0.350	0.666
3904	0.144	0.677	0.098	0.433	0.075	0.054	0.124	0.598
4508	0.057	0.027	0.012	0.026	0.064	0.036	0.063	0.073
537	0.038	0.022	0.017	0.035	0.090	0.215	0.050	0.060
15e	0.057	0.088	0.104	0.380	0.110	0.290	0.091	0.224
19b	0.268	0.480	0.331	0.453	0.533	0.420	0.336	0.481
14e	0.291	0.573	0.415	0.564	0.472	0.508	0.442	0.635

Non-neutralizing CD4i

Antibody	CRF250		C1080.c03		WITO4160.33		MGRM_002_8	
	SOSIP.664	gp120	SOSIP.664	gp120	SOSIP.664	gp120	SOSIP.664	gp120
A32	0.085	0.510	0.080	0.609	0.126	0.552	0.087	0.647
17b	0.059	0.050	0.009	0.078	0.099	0.348	0.418	0.157
E51	0.058	0.023	0.034	0.065	0.145	0.284	0.465	0.102

Non-neutralizing gp41

Antibody	CRF250		C1080.c03		WITO4160.33		MGRM_002_8	
	SOSIP.664	gp120	SOSIP.664	gp120	SOSIP.664	gp120	SOSIP.664	gp120
F240	0.007	-0.008	0.001	-0.004	0.014	-0.006	0.005	0.006

negative control

Antibody	CRF250		C1080.c03		WITO4160.33		MGRM_002_8	
	SOSIP.664	gp120	SOSIP.664	gp120	SOSIP.664	gp120	SOSIP.664	gp120
DEN3	-0.005	-0.023	-0.028	-0.016	-0.002	-0.027	-0.012	-0.001

Figure S1. Immunogen sequences, purification, and antibody reactivities, Related to Figures 1 and 2. A) Immunogen sequences C108.c03 (AE) (JN944660.1), CRF T-250 (AG) (EU513189.1), MGRM-002-8 (AG) (MF510462) and WITO.4130 (B) (AY835451.1) gp140 ectodomain regions (20-664 HXB2 numbering), were adapted to the SOSIP format by replacing the REKR furin cleavage site with R6 (RRRRRR), and by making the following mutations: A501C, I559P, T605C (red rectangles). The tissue-type plasminogen activator signal sequence was used and constructs with or without a C-terminal 8x His were made. The sequence was cloned into the multiple cloning site of the phCMV expression vector under control of the ubiquitously active CMV promoter. **B)** Size exclusion chromatography (SEC) elution volumes (280nm absorbance) for PGT145 purified SOSIP trimers and their corresponding SDS-PAGE reducing and non-reducing gels. S200 10/30 columns were equilibrated with PBS before injecting PGT145 eluted SOSIP from a 500ul injection loop. Trimer fractions were pooled, aliquoted, and frozen at -80°C. **C)** SOSIP trimers or sequence-matched gp120 proteins binding to mAbs are arranged by epitope. Values are given as response units (RUs) obtained using Biolayer Interferometry.10ug/ml of mAb is bound to anti-human IgG Fc sensors and maximum binding after 60s is recorded in 300nM SOSIP or 500nM gp120. Color scale is red to green from higher to lower binding.

A

WITO (immunogen: WITO at B1, B2, B3C; B4C: mixed)

ELISA antigen	animal	Pre	B1	B2	B3C	B4C
SOSIP: WITO (B) capture	P3899:	>16	7.18	0.13	0.10	0.73
	P3900:	>16	>16	0.56	0.06	0.89
	P4354:	>16	>16	0.11	0.09	1.53
	P4355:	>16	>16	0.19	0.19	1.34
SOSIP: C108 (AE) capture	P3899:	>16	>16	8.47	2.32	10.97
	P3900:	>16	>16	>16	2.38	10.71
	P4354:	>16	>16	5.82	2.30	14.06
	P4355:	>16	>16	>16	7.38	>16
SOSIP: T250 (AG) capture	P3899:	>16	>16	>16	7.46	>16
	P3900:	>16	>16	>16	2.40	>16
	P4354:	>16	>16	5.11	2.27	>16
	P4355:	>16	>16	9.65	6.22	>16
SOSIP: MGRM8 (AG) capture	P3899:	>16	>16	9.48	1.77	9.18
	P3900:	>16	>16	15.97	0.94	4.75
	P4354:	>16	>16	3.87	1.03	10.82
	P4355:	>16	>16	5.12	1.91	6.84
gp120: WITO (B) direct	P3899:	>16	>16	0.255	0.1185	0.8298
	P3900:	>16	>16	0.97	0.15	2.26
	P4354:	>16	>16	0.32	0.06	2.47
	P4355:	>16	>16	0.28	0.30	2.75
gp120: C108 (AE) direct	P3899:	>16	>16	>16	2.89	>16
	P3900:	>16	>16	>16	3.54	>16
	P4354:	>16	>16	13.44	0.36	>16
	P4355:	>16	>16	>16	>16	>16
gp120: T250 (AG) direct	P3899:	>16	>16	>16	0.80	>16
	P3900:	>16	>16	12.07	1.02	>16
	P4354:	>16	>16	>16	2.36	>16
	P4355:	>16	>16	>16	8.98	>16
gp120: MGRM8 (AG) direct	P3899:	>16	>16	>16	5.20	>16
	P3900:	>16	>16	>16	7.47	>16
	P4354:	>16	>16	>16	1.15	>16
	P4355:	>16	>16	>16	>16	>16
V3: autologous direct	P3899:	>16	>16	1.57	6.06	>16
	P3900:	>16	>16	3.64	3.92	>16
	P4354:	>16	>16	1.20	7.28	>16
	P4355:	>16	>16	1.37	11.71	>16

C108

ELISA antigen	animal	Pre	B1	B2	B3C	B4C
SOSIP: WITO (B) capture	P3901:	>16	>16	3.05	0.60	5.38
	P3902:	>16	>16	1.86	0.30	1.67
	P3903:	>16	>16	0.79	0.55	5.09
	P3912:	>16	>16	9.04	0.78	7.41
SOSIP: C108 (AE) capture	P3901:	>16	>16	0.26	0.22	2.31
	P3902:	>16	>16	0.85	0.29	1.45
	P3903:	>16	>16	0.25	0.26	2.63
	P3912:	>16	>16	2.49	0.30	3.44
SOSIP: T250 (AG) capture	P3901:	>16	>16	7.41	2.60	>16
	P3902:	>16	>16	8.17	2.03	7.24
	P3903:	>16	>16	2.08	1.77	>16
	P3912:	>16	>16	>16	3.15	>16
SOSIP: MGRM8 (AG) capture	P3901:	>16	>16	4.81	0.91	5.81
	P3902:	>16	>16	4.73	0.60	3.08
	P3903:	>16	>16	1.51	0.86	6.55
	P3912:	>16	>16	>16	0.99	7.36
gp120: WITO (B) direct	P3901:	>16	>16	>16	2.06	>16
	P3902:	>16	>16	>16	2.64	>16
	P3903:	>16	>16	14.97	8.22	>16
	P3912:	>16	>16	>16	7.37	>16
gp120: C108 (AE) direct	P3901:	>16	>16	3.13	0.49	5.81
	P3902:	>16	>16	0.86	0.54	4.14
	P3903:	5.69	8.20	1.13	0.58	10.83
	P3912:	>16	>16	1.10	0.10	3.94
gp120: T250 (AG) direct	P3901:	>16	>16	>16	>16	>16
	P3902:	>16	>16	>16	8.30	>16
	P3903:	>16	>16	>16	>16	>16
	P3912:	>16	>16	>16	>16	>16
gp120: MGRM8 (AG) direct	P3901:	>16	>16	>16	>16	>16
	P3902:	>16	>16	>16	>16	>16
	P3903:	>16	>16	>16	>16	>16
	P3912:	>16	>16	>16	>16	>16
V3: autologous direct	P3901:	>16	>16	7.39	>16	>16
	P3902:	>16	>16	4.95	>16	>16
	P3903:	>16	>16	1.21	>16	>16
	P3912:	>16	>16	>16	>16	>16

CRF250

ELISA antigen	animal	Pre	B1	B2	B3C	B4C
SOSIP: WITO (B) capture	P3906:	>16	>16	>16	0.15	1.08
	P3907:	>16	7.80	1.30	0.53	4.07
	P3910:	>16	>16	1.03	0.64	3.56
	P3898:	>16	12.62	1.27	2.76	9.94
SOSIP: C108 (AE) capture	P3906:	>16	>16	>16	3.76	12.91
	P3907:	>16	>16	>16	5.06	>16
	P3910:	>16	>16	10.27	3.36	10.80
	P3898:	>16	>16	8.37	>16	>16
SOSIP: T250 (AG) capture	P3906:	>16	>16	9.62	0.25	1.44
	P3907:	>16	11.25	0.86	0.69	3.91
	P3910:	>16	>16	0.73	0.62	3.39
	P3898:	>16	15.08	0.55	1.01	3.43
SOSIP: MGRM8 (AG) capture	P3906:	>16	>16	15.05	0.20	1.00
	P3907:	>16	11.36	1.01	0.44	2.22
	P3910:	>16	>16	0.56	0.45	2.55
	P3898:	>16	>16	0.85	1.88	6.31
gp120: WITO (B) direct	P3906:	>16	>16	>16	0.25	1.87
	P3907:	>16	>16	3.63	1.09	>16
	P3910:	>16	>16	1.72	0.62	8.08
	P3898:	>16	>16	3.18	2.33	>16
gp120: C108 (AE) direct	P3906:	>16	>16	>16	10.87	>16
	P3907:	>16	>16	>16	13.17	>16
	P3910:	>16	>16	>16	3.96	>16
	P3898:	>16	>16	4.37	5.40	>16
gp120: T250 (AG) direct	P3906:	>16	>16	>16	0.11	1.09
	P3907:	>16	>16	0.69	0.41	5.99
	P3910:	>16	>16	1.42	0.49	6.33
	P3898:	>16	>16	0.31	0.66	>16
gp120: MGRM8 (AG) direct	P3906:	>16	>16	>16	0.19	1.60
	P3907:	>16	>16	13.12	1.29	>16
	P3910:	>16	>16	9.99	0.58	9.09
	P3898:	>16	>16	10.77	15.94	>16
V3: autologous direct	P3906:	>16	>16	0.51	0.44	4.56
	P3907:	>16	0.65	0.29	1.80	>16
	P3910:	>16	1.56	0.15	0.78	5.65
	P3898:	>16	3.27	0.27	3.43	>16

MGRM8

ELISA antigen	animal	Pre	B1	B2	B3C	B4C
SOSIP: WITO (B) capture	P3904:	>16	>16	0.68	0.21	2.44
	P3905:	>16	>16	0.60	0.30	1.95
	P3909:	14.45	9.81	0.20	0.34	3.39
	P3911:	>16	>16	0.54	0.61	4.62
SOSIP: C108 (AE) capture	P3904:	>16	>16	3.03	1.35	11.06
	P3905:	>16	>16	4.68	1.56	6.31
	P3909:	>16	>16	6.10	1.80	14.72
	P3911:	>16	>16	9.01	3.19	>16
SOSIP: T250 (AG) capture	P3904:	>16	14.49	0.76	0.66	5.82
	P3905:	>16	>16	0.72	0.64	4.05
	P3909:	>16	6.42	0.15	0.27	1.51
	P3911:	>16	>16	0.13	0.15	0.87
SOSIP: MGRM8 (AG) capture	P3904:	>16	9.52	0.13	0.10	0.87
	P3905:	>16	>16	0.09	0.09	0.71
	P3909:	>16	6.42	0.15	0.27	1.51
	P3911:	>16	>16	0.13	0.15	0.87
gp120: WITO (B) direct	P3904:	>16	>16	1.08	0.27	3.97
	P3905:	>16	>16	1.03	0.31	1.76
	P3909:	>16	>16	0.30	0.35	4.83
	P3911:	>16	>16	0.81	0.85	12.37
gp120: C108 (AE) direct	P3904:	>16	>16	1.21	0.23	3.68
	P3905:	>16	>16	0.76	0.17	0.91
	P3909:	>16	>16	1.24	0.51	7.82
	P3911:	>16	>16	1.96	0.92	>16
gp120: T250 (AG) direct	P3904:	>16	>16	9.20	2.35	>16
	P3905:	>16	>16	3.64	0.78	8.64
	P3909:	>16	>16	2.47	1.15	>16
	P3911:	>16	>16	4.48	3.48	>16
gp120: MGRM8 (AG) direct	P3904:	>16	>16	0.28	0.21	4.52
	P3905:	>16	>16	1.10	0.48	2.67
	P3909:	>16	>16	1.07	0.58	11.38
	P3911:	>16	>16	1.49	0.89	11.82
V3: autologous direct	P3904:	>16	>16	0.70	5.05	>16
	P3905:	>16	>16	0.48	2.50	10.44
	P3909:	>16	>16	0.14	12.17	>16
	P3911:	>16	>16	0.56	4.22	>16

Mixed (cocktail) immunogen group

ELISA antigen	animal	Pre	B1	B2	B3C
SOSIP: WITO (B) capture	P5669:	>16	15.19	0.41	0.36
	P5670:	>16	>16	0.23	0.12
	P5671:	>16	>16	0.24	0.26
	P5672:	>16	15.19	0.84	0.15
SOSIP: C108 (AE) capture	P5669:	>16	>16	1.95	0.50
	P5670:	>16	>16	0.62	0.16
	P5671:	>16	>16	1.74	0.55
	P5672:	>16	>16	3.16	0.32
SOSIP: T250 (AG) capture	P5669:	>16	>16	1.24	1.16
	P5670:	>16	15.43	0.77	0.35
	P5671:	>16	>16	0.83	1.02
	P5672:	>16	>16	2.63	0.32
SOSIP: MGRM8 (AG) capture	P5669:	>16	>16	0.58	0.42
	P5670:	>16	>16	0.68	0.17
	P5671:	>16	>16	0.61	0.62
	P5672:	>16	>16	1.43	0.22
gp120: WITO (B) direct	P5669:	>16	>16	0.40	0.45
	P5670:	>16	>16	0.63	0.29
	P5671:	>16	>16	0.44	0.22
	P5672:	>16	>16	1.20	0.16
gp120: C108 (AE) direct	P5669:	>16	>16	2.78	2.02
	P5670:	>16	>16	0.89	0.00
	P5671:	>16	>16	3.00	1.43
	P5672:	>16	>16	8.10	1.00
gp120: T250 (AG) direct	P5669:	>16	>16	1.67	1.66
	P5670:	>16	>16	0.77	0.53
	P5671:	>16	>16	2.24	1.22
	P5672:	>16	>16	4.65	0.66
gp120: MGRM8 (AG) direct	P5669:	>16	>16	10.09	4.35
	P5670:	>16	>16	8.58	1.04
	P5671:	>16	>16	6.48	1.24
	P5672:	>16	>16	15.34	0.94

Sequential immunogen group (B1 CRF250, B2 WITO, B3C C108, B4C MGRM8)

ELISA antigen	animal	Pre	B1	B2	B3C	B4C
SOSIP: WITO (B) capture	P3893:	>16	>16	1.23	0.13	1.75
	P3894:	>16	8.71	0.21	0.06	1.27
	P3895:	>16	8.48	1.88	0.15	0.86
	P3896:	>16	>16	0.22	0.11	0.61
SOSIP: C108 (AE) capture	P3893:	>16	>16	>16	0.89	8.55
	P3894:	>16	>16	1.53	0.75	8.84
	P3895:	>16	>16	13.16	7.26	1.17
	P3896:	>16	>16	4.13	0.76	3.14
SOSIP: T250 (AG) capture	P3893:	>16	>16	3.27	0.63	6.10
	P3894:	>16	8.00	0.45	0.39	6.15
	P3895:	>16	10.59	5.37	0.79	4.06
	P3896:	>16	5.89	0.51	0.56	3.31
SOSIP: MGRM8 (AG) capture	P3893:	>16	>16	1.24	0.25	3.19
	P3894:	>16	>16	0.30	0.21	2.60
	P3895:	>16	6.96	3.40	0.39	1.76
	P3896:	>16	6.32	0.34	0.23	1.01
gp120: WITO (B) direct	P3893:	>16	>16	6.61	0.45	9.88
	P3894:	>16	>16	0.38	0.07	1.20
	P3895:	>16	>16	2.28	0.13	0.84
	P3896:	>16	>16	0.10	0.02	0.25
gp120: C108 (AE) direct	P3893:	>16	>16	92.96	1.26	21.74
	P3894:	>16	>16	2.21	0.50	5.52
	P3895:	>16	>16	11.15	0.73	7.68
	P3896:	>16	>16	1.74	0.30	1.47
gp120: T250 (AG) direct	P3893:	>16	>16	3.07	0.46	11.30
	P3894:	>16	>16	1.00	0.44	8.24
	P3895:	>16	>16	4.89	0.45	4.09
	P3896:	>16	>16	0.37	0.26	1.42
gp120: MGRM8 (AG) direct	P3893:	>16	>16	17.52	0.46	>16
	P3894:	>16	>16	1.92	0.44	9.80
	P3895:	>16	>16	14.37	0.33	8.83
	P3896:	>16	>16	0.84	0.18	0.93

:immunogen
:time point

B

EC50 (serum dilution factor)

virus neutralized	animal	Pre	B1	B2	B3C	animal	Pre	B1	B2	B3C	animal	Pre	B1	B2	B3C
WITO (B) tier 2	1(P3899) -	<20	<20	60.8	246.1	1(P3901) -	<20	<20	<20	<20	1(P3910) -	<20	<20	<20	<20
	2(P3900) -	<20	<20	<20	57.98	2(P3902) -	<20	<20	<20	<20	2(P3906) -	<20	<20	<20	<20
	3(P4354) -	<20	<20	29.8	1742	3(P3903) -	<20	<20	<20	<20	3(P3907) -	<20	<20	<20	<20
	4(P4355) -	<20	<20	29.7	58.11	4(P3912) -	<20	<20	<20	<20					
C108 (AE) tier 2	1(P3899) -	<20	<20	<20	<20	1(P3901) -	<20	<20	8.51	243.7	1(P3910) -	<20	<20	<20	<20
	2(P3900) -	<20	<20	<20	<20	2(P3902) -	<20	<20	4.05	113	2(P3906) -	<20	<20	<20	24.75
	3(P4354) -	<20	<20	<20	<20	3(P3903) -	<20	<20	12.1	272.6	3(P3907) -	<20	<20	<20	20.81
	4(P4355) -	<20	<20	<20	<20	4(P3912) -	<20	<20	522	1181					
T250 (AG) tier 2	1(P3899) -	<20	<20	<20	<20	1(P3901) -	<20	<20	<20	<20	1(P3910) -	<20	<20	<20	138.8
	2(P3900) -	<20	<20	<20	<20	2(P3902) -	<20	<20	<20	<20	2(P3906) -	<20	20.6	656	372.8
	3(P4354) -	<20	<20	<20	<20	3(P3903) -	<20	<20	<20	<20	3(P3907) -	<20	<20	31.2	33.45
	4(P4355) -	<20	<20	<20	<20	4(P3912) -	<20	<20	<20	<20					
MGRM8 (AG) tier 2	1(P3899) -	<20	<20	<20	<20	1(P3901) -	<20	<20	<20	<20	1(P3910) -	<20	<20	<20	<20
	2(P3900) -	<20	<20	<20	<20	2(P3902) -	<20	<20	<20	<20	2(P3906) -	<20	<20	<20	24.5
	3(P4354) -	<20	<20	<20	<20	3(P3903) -	<20	<20	<20	<20	3(P3907) -	<20	<20	21.7	<20
	4(P4355) -	<20	<20	<20	<20	4(P3912) -	<20	<20	<20	<20					

virus neutralized	animal	Pre	B1	B2	B3C	animal	Pre	B1	B2	B3C
WITO (B) tier 2	1(P3904) -	<20	<20	<20	<20	1(P3893) -	<20	<20	<20	<20
	2(P3905) -	<20	<20	<20	<20	2(P3894) -	<20	<20	<20	<20
	3(P3909) -	<20	<20	<20	<20	3(P3895) -	<20	<20	<20	<20
	4(P3911) -	<20	<20	<20	<20	4(P3896) -	<20	<20	<20	<20
C108 (AE) tier 2	1(P3904) -	<20	<20	<20	<20	1(P3893) -	<20	<20	<20	<20
	2(P3905) -	<20	<20	<20	<20	2(P3894) -	<20	<20	<20	<20
	3(P3909) -	<20	<20	<20	<20	3(P3895) -	<20	<20	<20	<20
	4(P3911) -	<20	<20	<20	<20	4(P3896) -	<20	<20	<20	<20
T250 (AG) tier 2	1(P3904) -	<20	<20	<20	<20	1(P3893) -	<20	<20	<20	<20
	2(P3905) -	<20	<20	<20	<20	2(P3894) -	<20	<20	<20	<20
	3(P3909) -	<20	<20	<20	<20	3(P3895) -	<20	<20	<20	<20
	4(P3911) -	<20	<20	<20	<20	4(P3896) -	<20	<20	<20	<20
MGRM8 (AG) tier 2	1(P3904) -	<20	23.6	46.8	95.1	1(P3893) -	<20	<20	<20	<20
	2(P3905) -	<20	22.4	99.9	105	2(P3894) -	<20	<20	<20	<20
	3(P3909) -	<20	<20	<20	38	3(P3895) -	<20	<20	<20	<20
	4(P3911) -	<20	<20	<20	<20	4(P3896) -	<20	<20	<20	<20

:immunogn
:time point

C

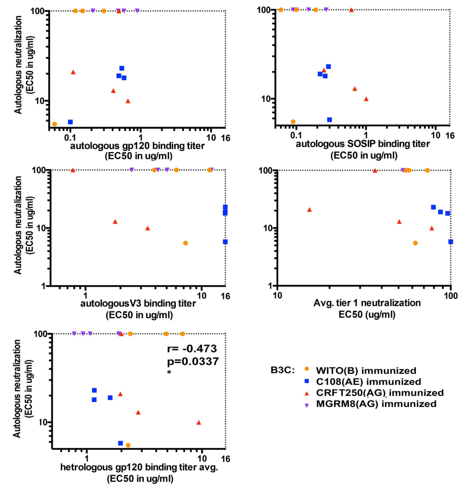

Figure S2. Binding titers, serum neutralization and data correlations for trimer-immunized rabbits, Relating to Figure 3. A) ELISA was used to measure binding titers to autologous and heterologous SOSIPs, gp120 and autologous V3 peptides. The table is organized with ELISA antigens given in the left column as colored boxes. Sample collection points for the different immunization groups (WITO: orange, C108: blue, CRF250: red, MGRM8: purple) are indicated across the top. Pre, B1, B2 and B3C correspond to the time points depicted in Figure 3A. Rabbit ID is given to the left of each immunization group. 50% maximum binding EC_{50}s in µg/ml of rabbit immune serum IgG are given. A darker grey colored box indicates higher binding titers. **B)** Autologous neutralization midpoint concentrations (IC_{50}s) as serum dilution factors calculated as was done for serum purified IgG in Figure 3B, for rabbits immunized with either WITO, C108, CRF250, MGRM8 or sequentially. The pseudovirus neutralized is on the far left. The rabbit ID is to the left of IC_{50}s organized by group across the page. Each sample time point is indicated at the top of the row. **C)** Autologous neutralization (IC_{50} in µg/ml rabbit immune serum IgG) at the B3C time point (when maximum autologous neutralization was observed) for the four groups immunized with individual SOSIPs were plotted against SOSIP, gp120 or V3 binding titers to identify possible correlates for the development of autologous neutralizing responses. Data points are colored according to the immunogen given. Heterologous binding titers are an average of the three heterologous titers.

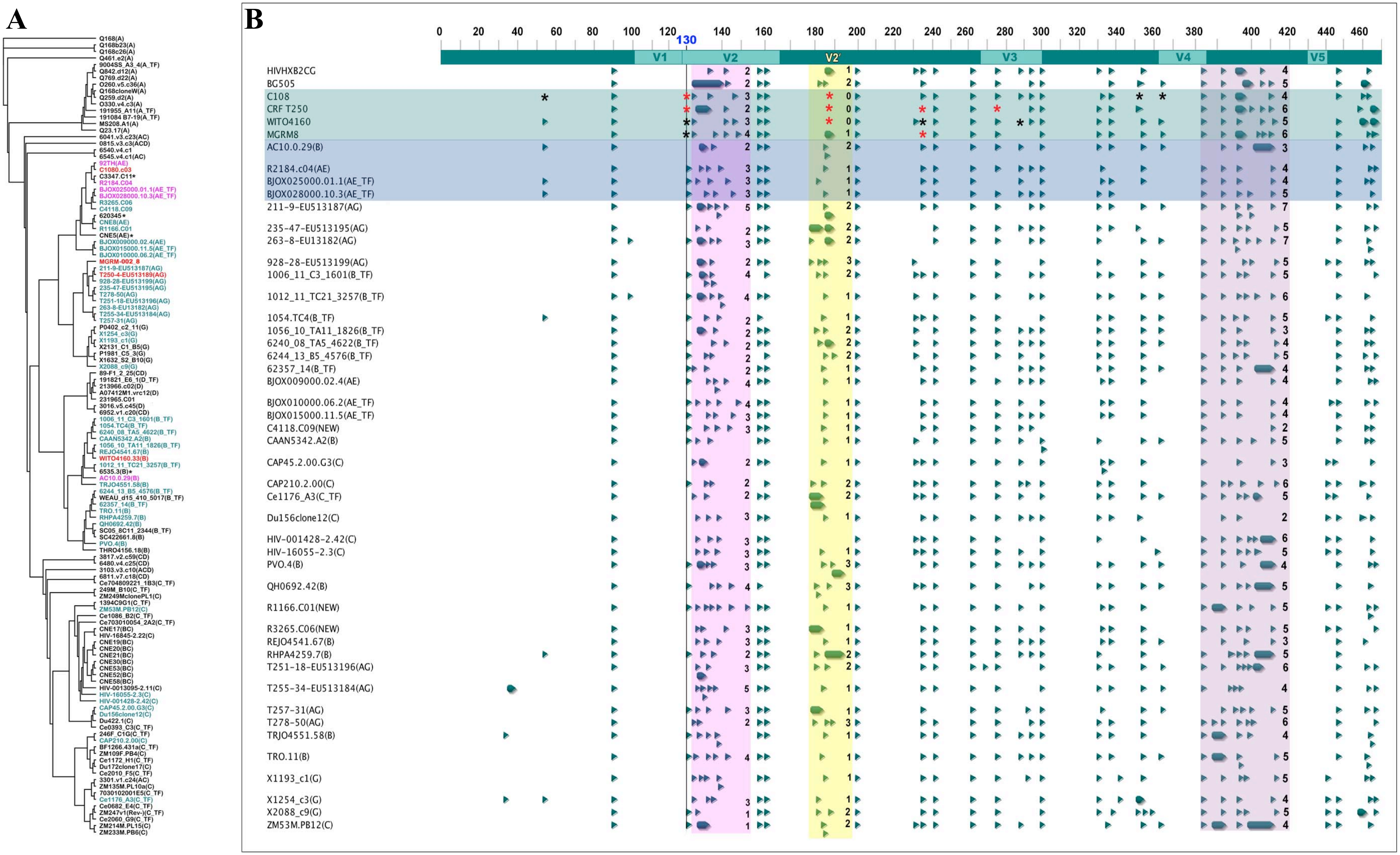

Figure S3. Phylogenic tree and glycan alignment of select tier 2 viruses. Relating to Figures 3-5. A) Phylogenic relationships of Tier 2 viruses screened for neutralizing breadth by rabbit immune serum IgG. Viruses screened were selected based on their similarity to immunogens by alignment (entire Env or V2 region) (colored). Immunogens sequences are in red. Heterologous viruses neutralized by rabbit P5672 are in pink. Viruses labeled with asterisks had poor infectivity and could not be used in neutralization assays. Viruses in cyan were tested but could not be neutralized. Four of the heterologous viruses neutralized are clade AE and cluster with C108, while one clade B (AC10) is most similar to the WITO immunogen, which failed to induce autologous neutralizing titers in this animal (P5672). **B)** Glycan alignment of gp120s from viruses that were screened for neutralizing breadth with rabbit immune serum IgG. Row at the top indicates region of the protein with numbering according to the HXB2 reference. Cyan arrows are NXT/S sites. Vertical columns of colored blocks have glycan sites within this sequence stretch summed to the right of the colored block. Black asterisk represent glycan hole sites that were filled and had no effect on rabbit immune serum IgG neutralization. Red asterisks indicate glycan hole sites that, when filled by adding an NXS/T site, attenuate/eliminate rabbit immune serum IgG neutralization (Figure 5).

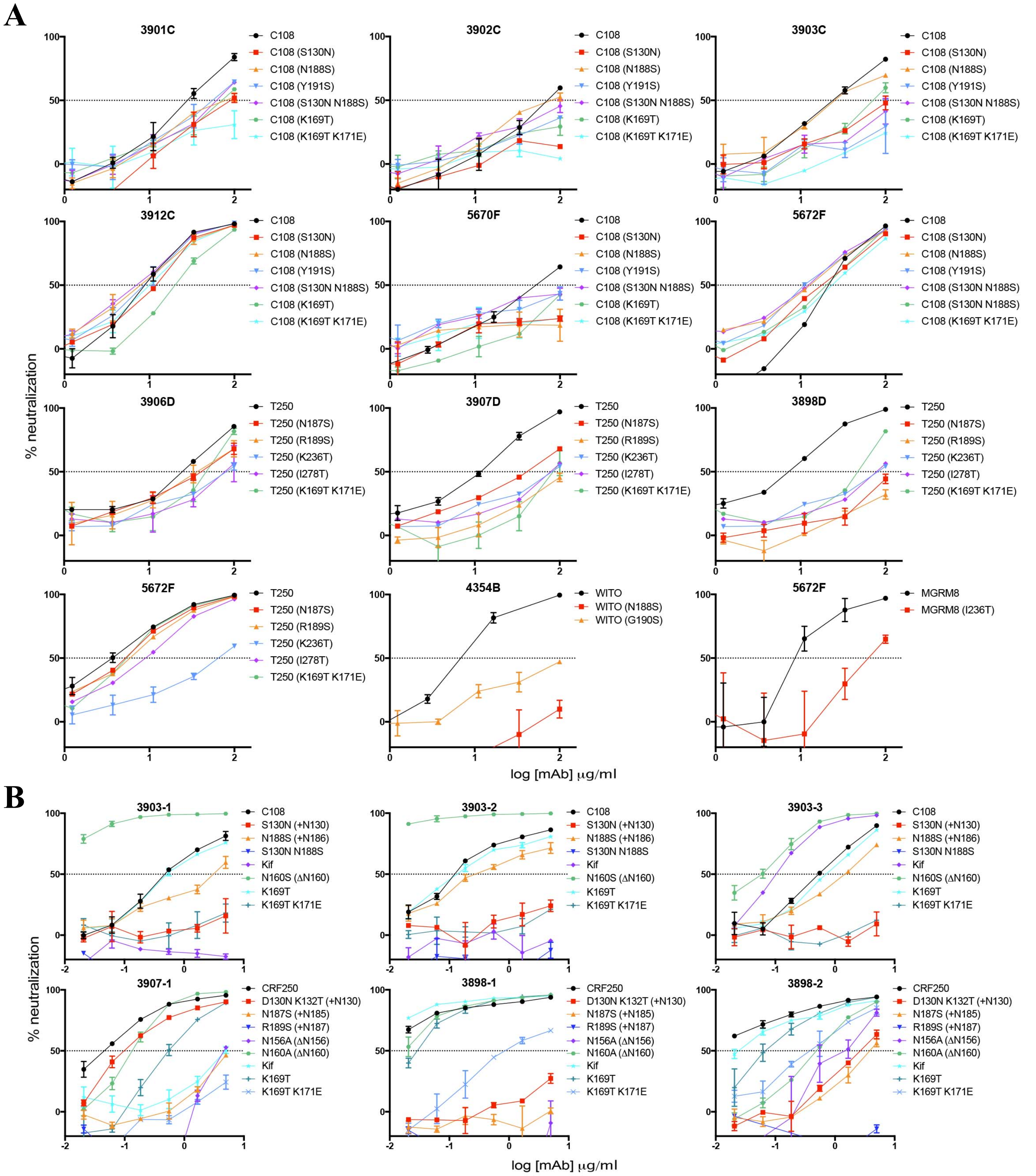

Figure S4. Neutralization titration curves using select virus mutants, Related to Figures 4 and 5. A) % neutralization is given as a function of the log of the polyclonal IgG concentration (in μg/ml) that was purified from immune serum at time B3 from the animal indicated in the title of the graph. IC_{50}s in Figure 4 are derived from these curves. **B)** % neutralization is given as a function of the mAb concentration in μg/ml for the mAbs indicated in the title of the graph. IC_{50}s in Figure 5 are derived from these curves. Viruses neutralized are given in the legends to the right of each graph. Virus mutations add PNG sites at 130, the V2' region, 234 or 276, or remove PNG sites at N156 or N160. Strand C mutants were also tested. Reduction or elimination of neutralization by select mutants helps to map neutralizing responses to specific epitopes on the wild type virus. Error bars represent SD between replicate data points.

A

B

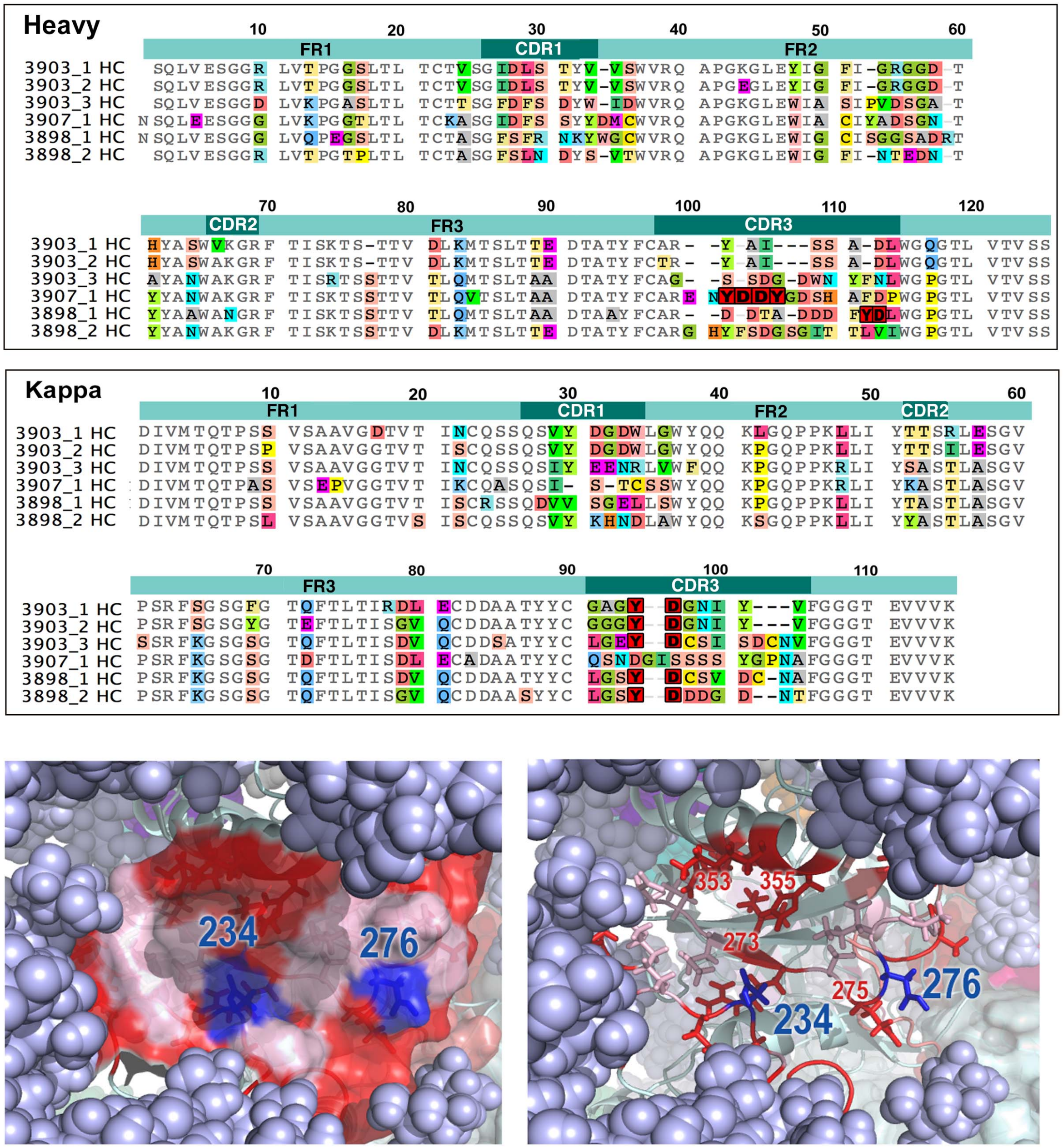

Figure S5. Alignment of rabbit neutralizing mAb sequences and depiction of an alternate glycan hole present in CRF250 and MGRM8 immunogens. Related to Figures 4 and 5. A) Heavy chain and kappa light chain VDJ and VJ genes respectively. Linear diagram above indicates framework and complementarity determining regions. Numbering is based on the consensus from all sequences. Positions at which sequences differ are colored by amino acid. YD motifs in CDR3s are represented in highlighted red blocks. **B)** A glycan hole proximal to the CD4 binding site and centered on glycans absent at 234 (CRF250/MGRM8), and 276 (CRF250). BG505 SOSIP trimer (PDB 4TVP) with glycans modeled in (also used in McCoy, LE et al. 2016) was used as a model to show a surface rendered image (on the left) or residue backbones and side chains (on the right), centered on the 234/276 site. Conserved residues between CRF250 and MGRM8 are shown in red, while residues that differ between them are in light pink. Surrounding modeled glycans are shown as violet and the location of this region in the context of the trimer and its position relative to the CD4 binding site is shown in Figure 4D.
