# Supplementary material for: Elicitation of Neutralizing Antibodies Targeting the V2 Apex of the HIV Envelope Trimer in a Wild-Type Animal Model
Source: Cell Rep. 2017 Oct 3;21(1):222–35. doi: 10.1016/j.celrep.2017.09.024 (PMC5640805; doi:10.1016/j.celrep.2017.09.024)
Supplement: Document S2. Article plus Supplemental Information [file mmc2.pdf]

Cell Reports

Article

Elicitation of Neutralizing Antibodies Targeting the V2 Apex of the HIV Envelope Trimer in a Wild-Type Animal Model

Graphical Abstract

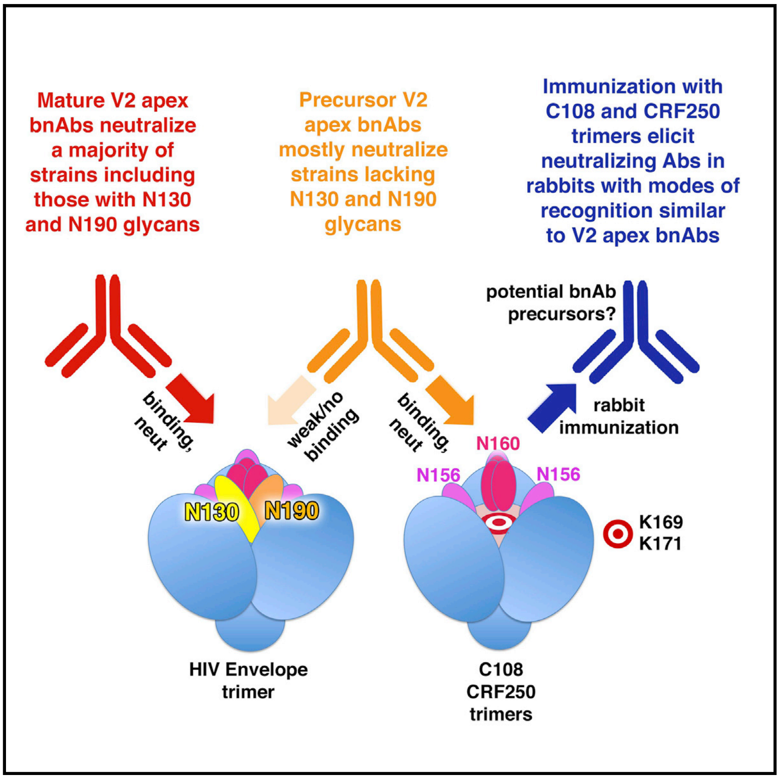

Authors

James E. Voss, Raiees Andrabi, Laura E. McCoy, ..., Max Crispin, Ian A. Wilson, Dennis R. Burton

Correspondence

burton@scripps.edu

In Brief

Voss et al. show that select V2-apex-focusing immunogens derived from bnAb precursor neutralization-sensitive HIV isolates can reproducibly elicit autologous neutralizing responses to components of the bnAb epitope, including K169/K171 and N156, in a wild-type animal model.

Highlights

- Precursor V2-apex bnAb neutralization-sensitive Envs possess a common glycan hole
- Sensitive Envs can be adapted to SOSIP format as V2-apex-focusing immunogens
- Immunogens elicit nAbs targeting critical components of the bnAb epitope in rabbits
- Select nAbs elicited show dependence on V2 lysines and glycans also important for bnAbs

Data and Software Availability

MF510462
MF510450
MF510451
MF510452
MF510453
MF510454
MF510455
MF510456
MF510457
MF510458
MF510459
MF510460
MF510461

Voss et al., 2017, Cell Reports *21*, 222–235
October 3, 2017 © 2017 The Author(s).
https://doi.org/10.1016/j.celrep.2017.09.024

CellPress

OPEN ACCESS CellPress

Cell Reports
Article

Elicitation of Neutralizing Antibodies Targeting the V2 Apex of the HIV Envelope Trimer in a Wild-Type Animal Model

James E. Voss,[1,2,3,9] Raiees Andrabi,[1,2,3,9] Laura E. McCoy,[1,2,3,6,9] Natalia de Val,[2,3,4] Roberta P. Fuller,[1,2,3] Terrence Messmer,[1,2,3] Ching-Yao Su,[1,2,3] Devin Sok,[1,2,3] Salar N. Khan,[1,3] Fernando Garces,[2,3,4] Laura K. Pritchard,[8] Richard T. Wyatt,[1,2,3] Andrew B. Ward,[2,3,4] Max Crispin,[1,3,8] Ian A. Wilson,[2,3,4,5] and Dennis R. Burton[1,2,3,4,7,10,*]

[1]Department of Immunology and Microbiology, The Scripps Research Institute, La Jolla, CA 92037, USA
[2]Scripps Center for HIV/AIDS Vaccine Immunology & Immunogen Discovery, The Scripps Research Institute, La Jolla, CA 92037, USA
[3]International AIDS Vaccine Initiative Neutralizing Antibody Center, The Scripps Research Institute, La Jolla, CA 92037, USA
[4]Department of Integrative Structural and Computational Biology, The Scripps Research Institute, La Jolla, CA 92037, USA
[5]Skaggs Institute for Chemical Biology, The Scripps Research Institute, La Jolla, CA 92037, USA
[6]Division of Infection and Immunity, Faculty of Medical Science, University College London, London WC1E 6BT, UK
[7]Ragon Institute of Massachusetts General Hospital, Massachusetts Institute of Technology, and Harvard, Cambridge, MA 02114, USA
[8]Oxford Glycobiology Institute, Department of Biochemistry, University of Oxford, Oxford OX1 3QU, UK
[9]These authors contributed equally
[10]Lead Contact
*Correspondence: burton@scripps.edu
https://doi.org/10.1016/j.celrep.2017.09.024

SUMMARY

Recent efforts toward HIV vaccine development include the design of immunogens that can engage B cell receptors with the potential to affinity mature into broadly neutralizing antibodies (bnAbs). V2-apex bnAbs, which bind a protein-glycan region on HIV envelope glycoprotein (Env) trimer, are among the most broad and potent described. We show here that a rare "glycan hole" at the V2 apex is enriched in HIV isolates neutralized by inferred precursors of prototype V2-apex bnAbs. To investigate whether this feature could focus neutralizing responses onto the apex bnAb region, we immunized wild-type rabbits with soluble trimers adapted from these Envs. Potent autologous tier 2 neutralizing responses targeting basic residues in strand C of the V2 region, which forms the core epitope for V2-apex bnAbs, were observed. Neutralizing monoclonal antibodies (mAbs) derived from these animals display features promising for subsequent broadening of the response.

INTRODUCTION

Neutralizing antibodies to HIV can protect against immunodeficiency viruses in animal models (Moldt et al., 2012; Pegu et al., 2014; Shingai et al., 2014), and their induction is seen as important for the generation of a successful HIV vaccine (Burton and Hangartner, 2016; Haynes and Mascola, 2017). The HIV envelope glycoprotein (Env) controls virus entry into target cells and is the sole target for neutralizing antibodies (nAbs) that block this process. Env is a membrane-anchored trimer of heterodimers $(gp120\text{-}gp41)_3$ that conceals much of its surface from antibody recognition using a shield of N-linked glycans (Behrens et al., 2016; Bonomelli et al., 2011; Lee et al., 2016; Stewart-Jones et al., 2016; Ward and Wilson, 2017). The antibody-accessible protein surfaces are highly variable in sequence and dynamic due to shifting N-linked glycan sequons, resulting in neutralizing responses that are mostly isolate specific (Moore et al., 2012; Sok et al., 2014). Despite these viral evasion strategies, broadly neutralizing antibodies (bnAbs) can emerge after years of affinity maturation during natural HIV infection in some individuals. These bnAbs, their affinity maturation from naive precursors, and their binding to HIV Env are being intensively studied in the hope that the information gathered can be used to design immunogens to elicit bnAbs, a process that has been termed "reverse vaccinology 2.0" (Burton, 2002, 2017; Rappuoli et al., 2016).

Several epitope regions on Env have been identified as targets for bnAbs (McCoy and Burton, 2017; Ward and Wilson, 2017; West et al., 2014). One such region is the trimer apex, comprising overlapping quaternary epitopes formed by the variable loop (V2) and N160 glycan from all three gp120 protomers (V2 apex) (Julien et al., 2013; McLellan et al., 2011; Pancera et al., 2013; Walker et al., 2009). In studies involving large cohorts of infected donors, bnAbs directed to the V2 apex emerge relatively frequently (21%–42% of all bnAbs) and early in infection, making the V2 apex region an attractive target for immunogen design (Georgiev et al., 2013; Landais et al., 2016; Walker et al., 2010). There are currently four prototype V2-apex bnAbs, and each was derived from different HIV-infected donors (PG9, CH01, PGT145, and CAP256.09) (Bonsignori et al., 2011; Doria-Rose et al., 2014; Walker et al., 2009, 2011). All four prototypes bind to a core epitope involving a lysine-rich β strand "C" (basic patch) in the V2 domain (HXB2 168–171) and N-linked glycans, particularly at residue 160 (N160) and, to a somewhat lesser extent, residue 156 (N156) (Andrabi et al., 2015; Gorman et al., 2016; McLellan et al., 2011; Pancera et al., 2013). The structural feature that allows

 © 2017 The Author(s).
This is an open access article under the CC BY license (http://creativecommons.org/licenses/by/4.0/).

OPEN ACCESS
CellPress

A	Subtype	PG9 iGL	CH01 iGL	CAP256 UCA	PGT145 iGL	K-rich	N130	N156	N160	(N) 183-191	(N) V1V2	V1 a.a. length	V2 a.a. length	V1' (HXB2 = 130-140)	V2' (HXB2 = 183-191)
HXB2	B	-	-	-	-	RGKVQK	-	+	+	+	5	27	39	KCTDLK--------------NDTNT	PIDN--------DTTS--Y
CRF02_AG_250	AG	178	<1.6	>100	>100	RDKKKK	-	+	+	-	4	22	39	DCQAFNS-----------SSHTNSS	QIN--------KNGRQ--Y
C1080.c03	AE	230	25	>100	>100	RDKKQK	-	+	+	-	5	28	38	SCTNVTLT------------NVNYT	QME-NK-----NS-----Y
WITO4160.33	B	182.0	<1.6	>100	>100	RDKIQK	-	+	+	-	5	25	41	HCTNVTI-------------SSTNG	PIEGKN----TNTG----Y
MGRM_BF_002_8	BF	>400	159	>100	>100	RDKKQK	-	+	+	+	7	34	39	ICSNVTSG-------SNVTSGSNVT	PMD--------NKSEE--Y
ZM233.PB6	C	2.0	3.4	>100	>100	RDKKRK	-	-	+	++	5	17	41	DCS--TYNNT-----------HNIS	PLTNSS-----NTTN---Y
16055-2.3	C	78	>400	>100	>100	RDKKQK	-	+	+	+	6	24	43	ECRQVNTTNAT--------SSVNVT	PLEEE---RKGNSS---KY
MGRM_AG_010_14	AG	40	>100	>100	>100	RDKRRK	+	+	+	++	7	23	46	NCSNSYN-----------TSKVNIT	QINEGKNNSNNNNTQ---Y
Q23.env17	A	44	<1.6	>100	>100	RDKRQK	-	+	+	-	4	19	40	HCTNVTS---------------VNT	PIN-EN--Q-GS-E----Y
MGRM_Chronic_B_014_2	B	>100	>100	>100	>100	RGKIQK	+	+	+	+	6	28	38	NCTDEVG-------------NTTNT	PMDN-------DTN----Y
ZM109F.PB4	C	>100	>100	>100	>100	KDRKQK	+	-	+	++	6	17	44	NCTSPAA--------------HNES	PLSSSD-NSSNSS----LY
CAP45.2.00.G3	C	>100	>100	>100	>100	RDKKQK	-	+	+	+	5	17	44	RCTNAT---------------INGS	PLNKNS----PSGNSS-EY
6535.3	B	>100	>100	>100	>100	GDKRQK	-	+	+	+	8	29	39	DCTDLNN-------------TTNTN	SIEN-------TSSS---Y
BG505.W6M.ENV.C2	A	>400	>400	>100	>100	RDKKQK	-	+	+	++	6	19	47	QCT---------------NVTNNIT	QINENQGNRSNNSNKE--Y
92RW020	A	>100	>100	>100	>100	KDKKQQ	-	+	+	+	5	15	41	DCN---------------ATASNVT	QINEK-----NETDK---Y
1012_11_TC21_3257	B	>100	>100	>100	>100	RDKVQK	+	+	+	+	8	30	39	NCTDVTN-------------ATNIN	PITNE------S-SK---Y
JRCSF	B	>100	>100	>100	>100	RNKVQK	-	+	+	+	5	25	40	NCKDVN--------------ATNTT	PIDNK-----NNTK----Y
ZM197M.PB7	C	>100	>100	>100	>100	RDRKTK	+	-	+	++	9	39	48	NCSDATSNTT-----------KNAT	PLEEEK-NSSSKNSSYKEY
ZM53M.PB12	C	>100	>100	>100	>100	RDKKKQ	+	+	+	+	6	15	41	NCSKLN----------------NAT	PLD-----GRNNSS---EY
C3347.c11	AE	>100	>100	>100	>100	KDKKQK	+	+	+	+	7	32	42	NCTKANLTSDT--------TNRTTG	PIKGNE----NSSG---EY
0260.v5.c36	A	>100	>100	>100	>100	RDKRQK	+	-	+	++	8	34	42	NCTDYKGNYTNYNYTKNNGTGHNIT	QINSSQ---TNSSE----Y
HIV-001428	C	>400	>400	>100	>100	RDKKQK	-	+	+	-	5	27	46	ECTQVNAT------------QGNTT	PLERENRGDSNSASK---Y

B

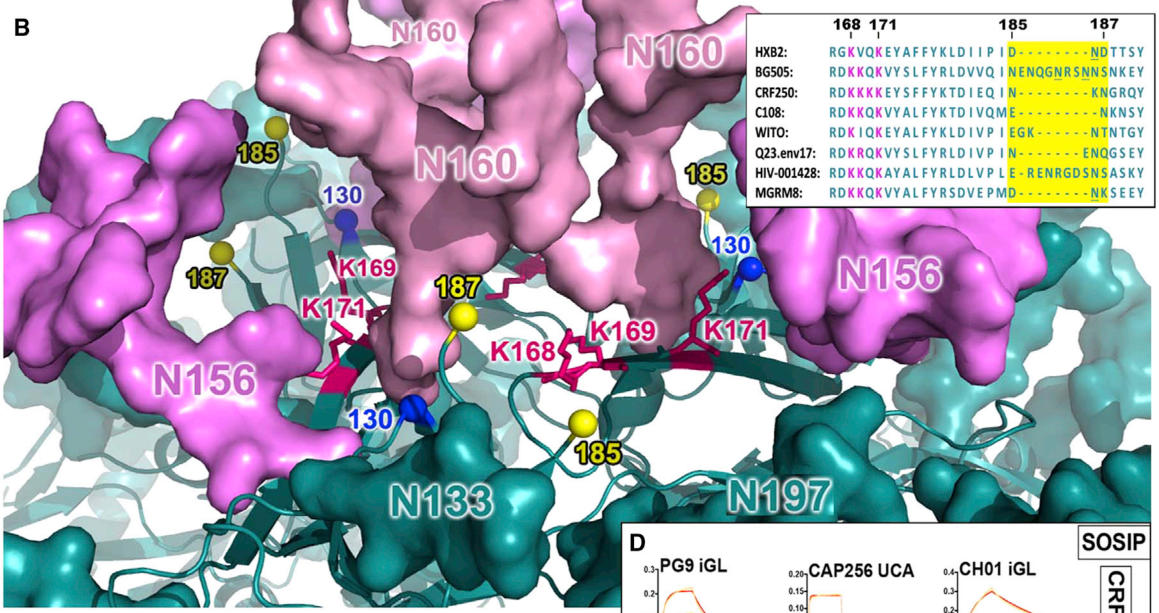

C	Subtype	PG9	PG9 iGL	CH01	CH01 iGL	N130	(N) 183-191
CRF02_AG_250	AG	<0.02	178	<0.02	<1.6	-	-
CRF02 (D130N, Q132T)		<0.02	340	0.65	>400	+	-
CRF02 (R189S)		<0.02	>400	0.24	181	-	+
C1080.c03	AE	<0.02	230	<0.02	25	-	-
C108(S130N)		0.03	>400	>5.0	>400	+	-
C108(Y191S)		0.02	295	0.43	>400	-	+
WITO4160.33	B	<0.02	182.0	<0.02	<1.6	-	-
WITO(S130N)		0.07	>400	0.43	>400	+	-
WITO(Y191S)		0.02	396.0	0.04	2.0	-	+
BG505.W6M.ENV.C2	A	0.03	>400	0.50	>400	-	++
BG505 (S183N N184S)		<0.02	>400	0.02	156	-	-

D

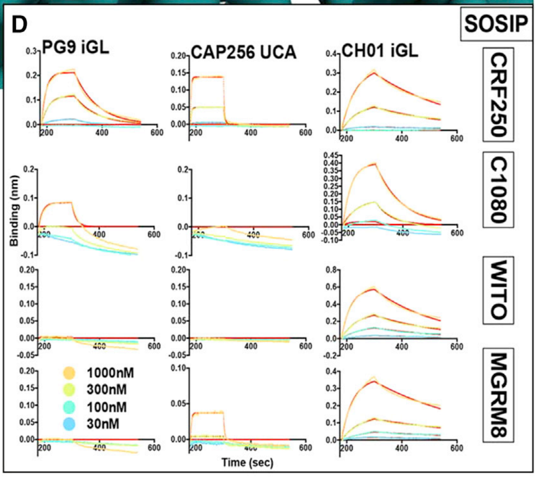

(legend on next page)

antibody penetration through the glycan shield to contact the V2 basic patch is an unusually long heavy-chain complementarity-determining region 3 (CDRH3) loop, which for PG9 and CAP256.09 makes contact with strand-C lysines through a conserved germline-encoded tripeptide YYD motif originating from the same D-gene (Andrabi et al., 2015; McLellan et al., 2011; Pancera et al., 2013).

We and others have previously described viruses that are neutralized by inferred precursors (iPs) of prototype apex bnAbs (Andrabi et al., 2015; Gorman et al., 2016). It is logical that the Env immunogens derived from these viruses might effectively expand V2-apex bnAb precursor lineages in humans. We showed that one of these Env sequences could be adapted to produce recombinant soluble near-native SOSIP.664 trimers (referred to hereafter as SOSIPs). Here, we describe the identification of a larger panel of four SOSIPs derived from V2-apex bnAb iP-sensitive viruses, which have antibody-binding profiles representative of functional Env spikes. Sequence analysis revealed that three of these Envs lack predicted N-linked glycosylation (PNG) sites at position 130 (HXB2 numbering) and within the V2 hypervariable region (designated the V2′, HXB2 position 183–191) that constitute a rare "glycan hole" at the V2 apex, as has been described at other Env locations (Crooks et al., 2015; Klasse et al., 2016; McCoy et al., 2016). Incorporation of PNG sites to fill this hole disrupted neutralization by iP antibodies, suggesting this glycan hole may aid the elicitation of bnAbs to this general region.

To assess the ability of our immunogens to focus responses to the V2 apex region, we immunized rabbits using all four V2-apex bnAb iP-binding SOSIPs given individually, as a mixture, or sequentially. Marked differences were found among the trimers in their ability to elicit autologous nAbs. The dominant target of these responses was the same glycan hole at the V2 apex that was required for neutralization by the iP versions of prototype V2-apex bnAbs. One trimer immunogen consistently elicited responses involving strand-C basic patch residues, which are also key contacts for prototype bnAbs. Therefore, it appears that this hole in the glycan shield may indeed improve exposure of the V2-apex bnAb region to neutralizing responses. Encouragingly, we also report the elicitation of neutralizing responses targeting several heterologous tier 2 viruses in one animal that was immunized with a SOSIP trimer cocktail.

RESULTS

Glycan Holes Are a Major Determinant for V2-Apex bnAb iP Binding on Select Env-Based Trimers

HIV isolates screened for sensitivity to neutralization by iP versions of PG9, CH01, and PGT145 antibodies (Andrabi et al., 2015; Gorman et al., 2016) and the CAP256 unmutated common ancestor sequence (UCA) (Doria-Rose et al., 2014) are shown in Figure 1A. We compared Env sequences from eight iP-sensitive isolates to identify critical features that might facilitate neutralization. Strikingly, four of these Envs lacked PNG sites at locations in close proximity to key bnAb contacts, the strand-C lysines, suggesting the presence of a glycan hole at the V2 apex. These Envs are derived from C108.c03 (hereafter referred to as C108, clade AE), CRF-T250 (hereafter CRF250, clade AG), WITO4160.33 (hereafter WITO, clade B), and Q23.env17 (clade A), all neutralized by both PG9 and CH01 iP antibodies (Figure 1A).

The V2-apex glycan hole is modeled onto the BG505 SOSIP structure (Gristick et al., 2016), in Figure 1B to reveal absent PNG sites at position 130 (HXB2 numbering) and within a loop projecting apically from the V2 hypervariable region designated V2′ (HXB2 183–191). This loop is unstructured in the BG505 model (HXB2 185–187). The glycan at 130 (N130) is conserved in 56% of viruses from a 120-virus panel (Seaman et al., 2010) representing the global diversity of HIV. However, at least one PNG site is highly conserved in the V2′ loop (94% of viruses in the panel), so that viruses lacking glycans at both sites represent only ~4% of these isolates. To directly investigate the role of these glycans on V2-apex iP antibody binding, we incorporated PNG sites at N130 and/or within the V2′ region in three of these Envs and assessed their neutralizing sensitivity to the iPs. PNG sites added to at least one of these two positions either reduced or eliminated neutralizing activity of the iPs, with the effect being more prominent for N130 (Figure 1C). For BG505 (already lacking a PNG site at 130), the removal of two PNG sites within the V2′ region rendered this virus sensitive to neutralization by the CH01 iP (Figure 1C). HIV-001428, the only other virus from the 120-virus global panel lacking glycans at both these positions, was not neutralized by any iP (Figure 1A), suggesting that the absence of glycans at both 130 and within the V2′ is not sufficient for iP reactivity in some Env contexts. HIV-001428 has an especially long V2′ region (Figure 1B). This may obstruct iP access to important strand-C contacts. iP antibodies can tolerate glycans

Figure 1. Sensitivity of Select Envs to iP Versions of V2-Apex Prototype bnAbs

(A) Env iP neutralization table. HIV isolates neutralized by three prototype V2-apex bnAb iP Abs and the CAP256 UCA. Neutralization IC_{50} values against each pseudovirus are in micrograms per milliliter. Envelope features, including the strand-C lysine-rich basic patch sequence and V1 V2 regions, are also noted. The presence of PNG sites at specific sequences are marked by a plus (+) sign (two glycans are ++) and the total number of PNG sites in the V1V2 loop are given. NXT/S sequons are indicated by a bold red N in sequence alignments. Envs that were successfully generated as soluble native trimers are in blue font.

(B) Trimer V2-apex model. The BG505 trimer (PDB: 5T3X) highlighting residue 130 (blue spheres) and the base of the V2′ hypervariable region loop. Sequence variation in this region between select Envs is shown in the insert on the right. Residues between the last structured amino acids in the V2′ region loop (185 and 187; HXB2) are highlighted in yellow with NXS/T sequons underlined. 130 and V2′ sites lie close to each other and to the V2-apex bnAb core epitope, including the strand-C basic residues N156 and N160 (pink).

(C) iP antibody neutralization of V2-apex glycan variants. Shown are neutralization IC_{50} values (in micrograms per milliliter) for mature or iP PG9 and CH01 antibodies against virus variants with V2-apex PNG sites at 130 or within the V2′ region either added or removed.

(D) Biolayer interferometry (BLI) showing the binding and dissociation curves for PG9 and CH01 iPs and for CAP256 UCA to PGT145-purified SOSIPs. Binding was measured by incubation with the indicated concentrations of SOSIP (180–300 s) measured in delta (nanometers) or response units. Dissociation of SOSIP from the IgG in PBS buffer was further recorded.

at either or both of these positions in some Env sequence contexts, such as MGRM8, MGRM14, ZM233, and 16055 (Figure 1A). V1 or V2 lengths did not correlate with iP sensitivities (Figure 1A).

To investigate whether the iP-sensitive Envs could be used as immunogens to guide a V2-apex-specific neutralizing response, we first incorporated SOSIP.664-trimer-stabilizing modifications as previously described (Pugach et al., 2015; Sanders et al., 2013). Four of these Envs (CRF250, C108, WITO, and MGRM-002-8 [hereafter MGRM8]) (Figure S1A) produced soluble trimer when purified by PGT145 antibody (Ab) affinity chromatography. PGT145 enriches only native-like homogeneous trimer populations that are properly folded (Pugach et al., 2015). All SOSIP trimers eluted as single peaks with sizes consistent with the trimeric form by size exclusion chromatography (SEC) (Figure S1B).

Next, we assessed the binding of the PG9, CH01 iPs, and CAP256 UCA Abs to the soluble SOSIP trimers by biolayer interferometry (BLI). Consistent with the neutralization data, the iP Abs displayed significant binding to the soluble trimers (Figure 1D). Among the four SOSIPs, only the CRF250 trimer showed binding to all three iP Abs. The virus encoding this Env shares V2-apex sequence similarity with CAP256 superinfecting virus (CAP256.SU), a descendant of which likely triggered the CAP256 V2-apex bnAb lineage (Bhiman et al., 2015; Doria-Rose et al., 2014). CRF250 also has a pattern of neutralization sensitivity similar to CAP256.SU by the CAP256 bnAbs. While WITO was neutralized by both PG9 and CH01 iPs, binding of its SOSIP could only be detected for CH01, possibly reflecting subtle differences between the Env as a membrane-associated functional trimer on the viral surface and the PGT145-enriched SOSIP-stabilized recombinant trimers. In all, we demonstrate that a glycan hole at the V2 apex is associated with binding to iP versions of V2-apex bnAbs in three of four vulnerable HIV-1 isolates that could be successfully produced as soluble trimers. These trimers showed iP-binding properties mostly consistent with iP neutralization data.

The V2-Apex iP-Binding SOSIPs Form Homogeneously Folded Trimers and Display Native-like Properties

Next, we characterized the SOSIP trimers that bound V2-apex iPs, biophysically and antigenically, to determine their suitability as immunogens. PGT145-purified SOSIPs formed well-ordered, 3-fold symmetric trimers by negative-stain electron microscopy (EM) (Figure 2A), which were efficiently cleaved into gp120 and gp41 as assessed by SDS-PAGE under reducing conditions (Figure S1B). Enzymatically released and fluorescently labeled N-glycans were analyzed by hydrophilic interaction ultraperformance liquid chromatography (HILIC-UPLC) (Behrens et al., 2016; Pritchard et al., 2015) to determine the glycan composition for each trimer. The profiles were comparable to BG505 SOSIP expressed in 293T cells, with a large proportion of high-mannose glycans $Man_{8-9}GlcNAc_2$ (Man_{8-9}) and a smaller fraction of mannose $Man_{5-7}GlcNAc_2$ (Man_{5-7}) and complex glycans (Figure 2B).

Differential scanning calorimetry (DSC) was used to assess the thermal stability of the trimers and showed single unfolding peaks for CRF250, C108, and MGRM8 trimers with thermal denaturation midpoints (T_m) of 68.1°, 70.8°, and 65.1°, respectively. These thermostabilities are similar to that of BG505 SOSIP, which has a T_m of 68.1°C (Sanders et al., 2013). The WITO SOSIP trimer showed two melting peaks (T_m) at 57.7° and 66.9°, indicating the presence of an uncharacterized heterogeneous mixture of trimer populations (Figure 2C).

To evaluate the overall antigenic profile of the SOSIP trimers generated here, we used BLI to characterize the binding of trimers and their sequence-matched gp120 proteins to a panel of 85 Env-specific neutralizing and non-neutralizing Abs directed to a range of epitopes. These include antibodies targeting the V2 apex (n = 30), V3-glycan (n = 16), CD4-binding site (CD4bs) (n = 14), gp120-gp41 interface (n = 10), non-neutralizing V3 (n = 11), and CD4-induced (CD4i) (n = 3) and gp41 cluster-1 (n = 1) (Figures 2D and S1C). The trimers generally displayed strong reactivity with bnAbs. All four trimers showed robust binding to the V2-apex-directed and gp120-gp41 interface bnAb epitopes. Weak or no antibody binding to the corresponding sequence-matched gp120s was observed, consistent with the trimer-specific/preferring nature of bnAbs to these sites. V3-glycan bnAbs were also trimer preferring, with the exception of reactivity with MGRM8 Env, where they bound to trimer and gp120 equally. The trimer preference of V3-glycan bnAb PGT121 lineage intermediates has been described previously (Sok et al., 2013). CD4-binding-site bnAbs did not show a preference for trimers, and CRF250 showed unusually low antibody binding to this site. We included four non-neutralizing V2-apex-directed Abs (2909, C108G, 830A, and 697) to examine whether the glycan hole exposed non-neutralizing V2 epitopes. Only WITO and MGRM8 SOSIP trimers weakly bound 830A (Figure S1C). Non-neutralizing V3 reactivity was seen with all trimers. WITO showed V3 reactivity similar to that observed for its gp120 monomer. CRF250 and MGRM8 trimers showed reduced V3 reactivities compared with their respective gp120s. C108 trimer showed the lowest reactivity, with only three V3-specific monoclonal antibodies (mAbs) showing binding over 0.2 response units (RUs) (Figure 2D). However, binding to C108 gp120 was also lower than for other strains. Trimer V3 reactivities are consistent with some unfolding or breathing and exposure of V3 in a fraction of SOSIP molecules, as described for other HIV strains (Sanders et al., 2013). Additionally, the MGRM8 trimer showed reactivity with two CD4i mAbs, suggesting that it may be somewhat prone to partial unfolding or opening to the CD4-bound form (Figure 2D). We further tested the ability of these mAbs to neutralize viruses pseudotyped with Envs corresponding to the four SOSIP timers and observed a strong correlation between binding and neutralizing activity (Figure 2E). These data suggest that the four recombinant immunogens are in conformations representative of their native functional states on viral surfaces.

Immunization of Rabbits with V2-Apex iP-bnAb-Binding Soluble Trimers Elicits Autologous Neutralizing Responses to Varying Extents

To evaluate the immunogenicity of the iP-binding trimers and determine whether they could guide a V2-apex-focused neutralizing response, we chose New Zealand white rabbits (*Oryctolagus cuniculus*) as an animal model. This is because rabbits have previously induced well-characterized high-titer tier 2

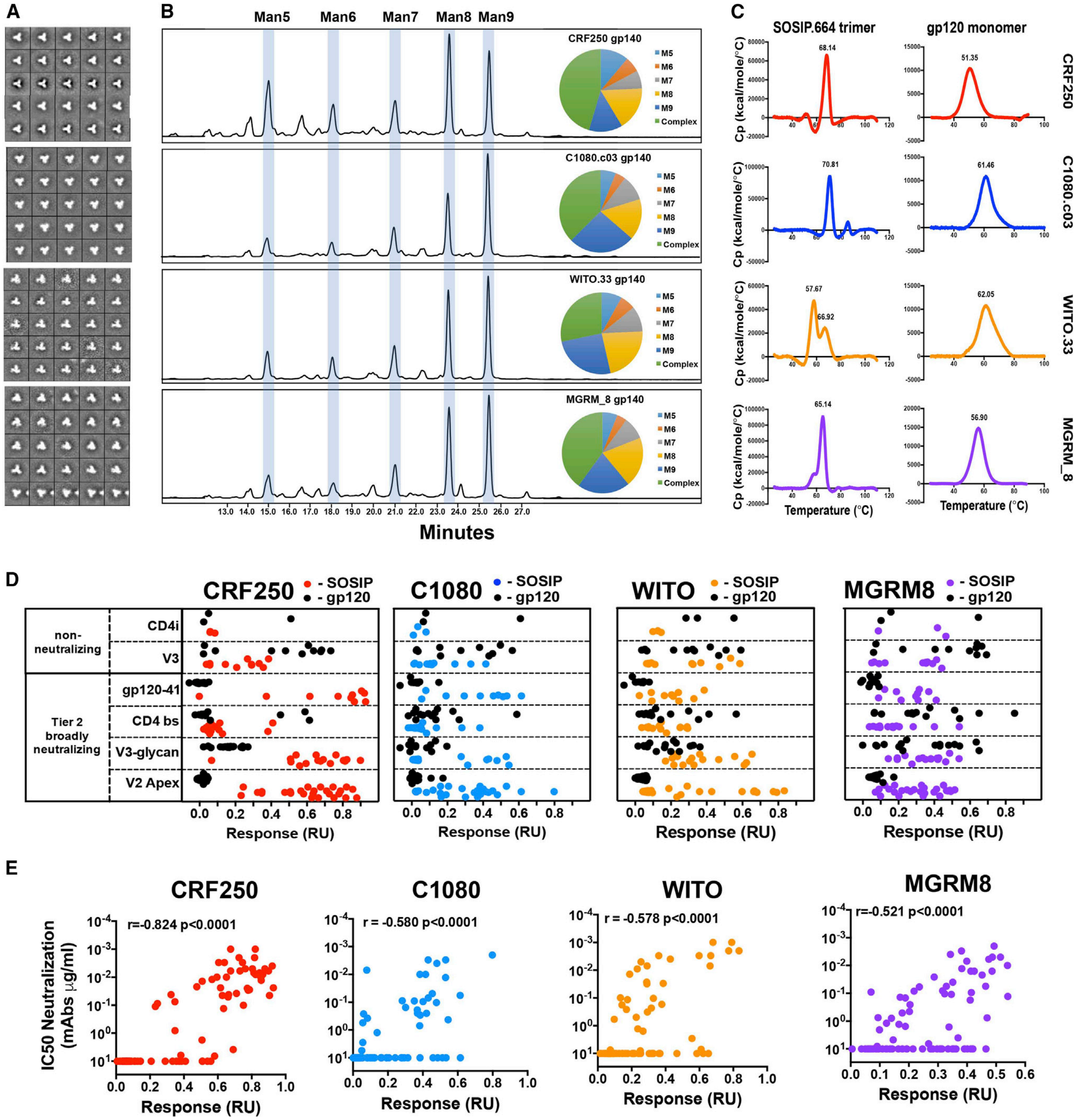

Figure 2. Biophysical and Antigenic Properties of iP-Binding Soluble Trimers

(A) Negative-stain EM. Class average, negative-stain EM images of CRF250, C108, WITO, and MGRM8 native trimers in descending order.

(B) SOSIP glycan composition. Elution time of enzymatically released N-glycans from SOSIP immunogens in HILIC-UPLC. Area of eluted peaks represents the quantity of a particular glycoform (indicated above the peak). Pie diagrams indicate glycoform ratios for each of the immunogens.

(C) Differential scanning calorimetry (DSC) showing the thermal stability of the SOSIP immunogens and their sequence-matched gp120s. Protein denaturation is measured in kilocal/mol/°C. Melting peak temperature (T_m) is indicated above the peak.

(D) mAb binding to SOSIPs. Maximal binding in response units (RUs) of 500 nM CRF250 (red), C108 (blue), WITO (orange), and MGRM8 (purple) SOSIPs (colored circles) as well as their sequence-matched gp120s (black circles) to a panel of 85 mAbs measured by BLI. The Abs are divided by epitope as indicated on the far left y axis. RU values are given in Figure S1C.

(E) Neutralization activities. Pseudovirus neutralization IC_{50} values plotted against SOSIP-binding response units show a strong correlation (two-tailed Spearman R).

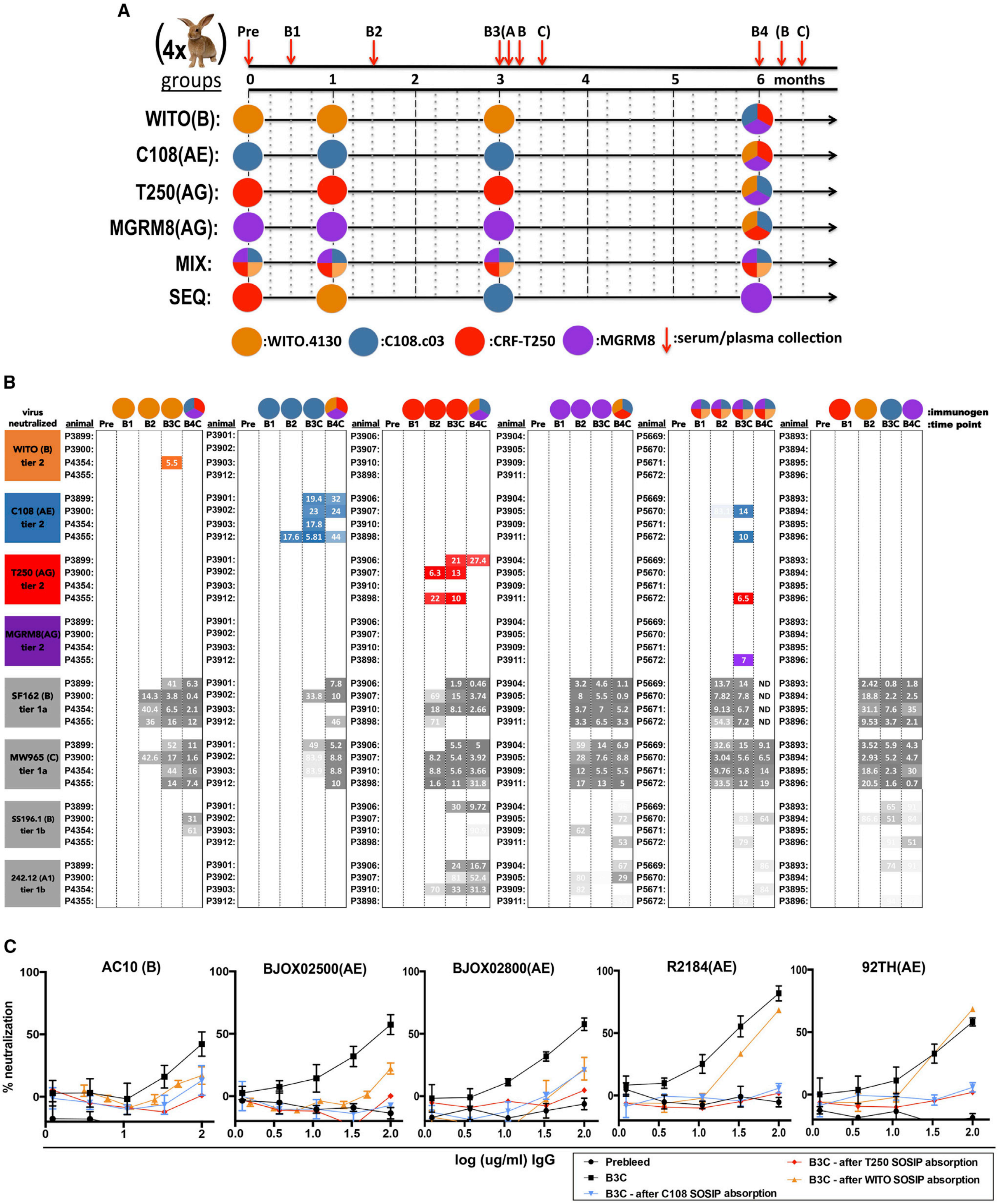

Figure 3. Rabbit Trimer Immunizations and Elicited Neutralizing Responses

(A) Immunization schedule. Six groups of 4 female New Zealand white rabbits were immunized with mixtures or individual SOSIPs as indicated by the colored circles. 50 μg total protein and 100 U Iscomatrix were given intramuscularly (IM) at 0, 1, 3, and 6 months. Serum or plasma was collected at times indicated by red arrows.

(legend continued on next page)

neutralization in response to other SOSIP immunogens (Klasse et al., 2016; Sanders et al., 2015). Further, rabbit and human repertoires share similar CDRH3 length frequencies and amino acid compositions (Lavinder et al., 2014), and this region is thought to be critical for V2-apex bnAb function.

Groups of four, 3-month-old, female rabbits were immunized with the individual SOSIP trimers, a cocktail of all four trimers, or with each trimer sequentially as depicted in Figure 3A. Each immunization contained 50 μg trimer given with 100 U Iscomatrix adjuvant injected intra-muscularly (IM). Animals were immunized at 0, 4, and 12 weeks. A final boost, unique to each group, was administered at 24 weeks post-prime. For this final boost, groups immunized with individual SOSIP trimers were given a cocktail of the three other heterologous trimers used in the study (total protein in the cocktail remained at 50 μg/dose); the cocktail-immunized group received a final injection of all four SOSIPs, and the sequentially immunized group received its final SOSIP trimer (Figure 3A). The sequential immunization protocol relied on the conserved gp41 sequence to provide adequate helper T cell epitopes, and SOSIP-binding titers from this group show prime-boost responses, suggesting that adequate T cell help was available for B cells responding to the variant immunogens (Figure S2A). Peripheral blood mononuclear cells (PBMCs) and sera were collected at the time points indicated in Figure 3A. Neutralization against the autologous virus was first assessed using rabbit immune sera (Figure S2B), but to allow quantification of the response (in micrograms per milliliter) of immunoglobulin G (IgG), all analyses were subsequently carried out using polyclonal-protein-A/G-purified IgG from serum.

Autologous neutralization peaked after the second boost as reported for BG505 SOSIP trimers in rabbits (Figure 3B) (Klasse et al., 2016; McCoy et al., 2016; Sanders et al., 2015). The individual SOSIP trimers appear to differ in their ability to raise autologous nAbs, although group sizes were small. For instance, C108 trimer reproducibly elicited strong autologous neutralization titers in 4 out of 4 animals, while CRF250, WITO, and MGRM8 trimers elicited autologous neutralization titers in 3 out of 4, 1 out of 4, and 0 out of 4 animals, respectively (Figure 3B).

In the SOSIP-mixture-immunized group, two animals developed autologous neutralizing titers against the C108 virus, and one of these rabbits (P5672) also developed neutralizing activity against two other immunogen-matched autologous viruses (CRF250 and MGRM8) (Figure 3B). Interestingly, this was the only animal in the study to develop high titers of nAbs to MGRM8 virus, and animals immunized with MGRM8 alone did not. In the sequential immunization group, no animal developed significant neutralizing responses to any of the four immunogens (Figure 3B).

Tier 1 viruses expose a number of epitopes that are hidden on tier 2 viruses and can be neutralized by antibodies that target relatively conserved regions and are therefore cross-reactive but not broadly neutralizing against primary isolates (Seaman et al., 2010). Animals in all the immunogen groups developed robust neutralization titers to tier 1a viruses from clades B and C, except for the C108-only group (Figure 3B), but there was no correlation between autologous neutralization and heterologous tier 1 neutralization (Figure S2C).

Eight Tier 2 Viruses Can Be Neutralized by Responses in One Animal Immunized with the Trimer Mix

All animals that showed autologous neutralizing activity after the second boost were screened for neutralizing breadth using a number of tier 2 pseudoviruses selected based on similarity with component immunogen Envs (either gp120 or V1 V2 sequence; Figure S3A). Animal P5672 from the cocktail-immunization group showed neutralizing activity against five heterologous, tier 2 viruses. These included AC10 (subtype-B), R2184.c03, BJOX028000, BJOX25000, and 92TH01 (subtype-AE) viruses (Figure 3C). P5672 neutralization activity against these isolates could be adsorbed by C108 and CRF250, but not WITO, SOSIP trimers (Figures 3C and S3A). These isolates did not lack a common glycan compared with the immunogens (Figure S3B). To investigate the effect of preexisting immunological memory on the elicitation of nAbs, animals originally exposed to only one of the four immunogens were boosted with a mix of all three heterologous trimers used in the study. No high-titer neutralizing responses were elicited to any SOSIPs in the final boost, suggesting these immunogens do not strongly recall preexisting potentially cross-reactive neutralizing responses (Figures 3A and 3B).

Serum-Binding Titers to Env Do Not Predict the Elicitation of Autologous Neutralizing Responses

Serum-antibody-binding titers to Env preparations were determined by ELISA. Rabbit IgG purified from serum was used to generate half maximal effective concentration (EC_{50}) values given in micrograms per milliliter of polyclonal purified IgG. We assessed binding to SOSIP trimers, monomeric gp120s, and V3 peptides (Figure S2A). Similar autologous-binding titers were elicited by all immunogens used in this study. Titers appeared after the first boost and improved only slightly after the second boost. This trend was observed in both SOSIP trimer and gp120 monomer ELISAs. Autologous-binding EC_{50} values are outlined by immunogen colored boxes in Figure S2A. There was no correlation between autologous gp120 or SOSIP trimer binding titer and autologous nAb responses (Figure S2C). There

(B) Autologous and tier 1 neutralization responses in immunized rabbits. Serum IgG from rabbit pre-bleeds or at 14 days post-immunization (B1, B2, B3C, and B4C) was assessed for neutralizing ability against the sequence-matched pseudovirus for the SOSIP immunogens used in this study (colored boxes) or tier 1 pseudoviruses (gray boxes) on the left side of the table. Data are arranged by immunization group from left to right, and the immunogen most recently given for the time point in each group is indicated across the top of the table by a colored circle. Pseudovirus neutralization by rabbit immune serum IgG is given as the IC_{50} (in micrograms per milliliter) of IgG purified from rabbit serum. Samples are indicated by rabbit identification numbers to the left of IC_{50} values. Higher neutralizing responses are colored.

(C) Neutralization of heterologous viruses by SOSIP mixture immunized rabbit, P5672. Virus neutralization as a function of rabbit immune serum IgG concentration is shown for five tier 2 clade AE viruses (92TH is an unconfirmed tier 2 virus), neutralized by the cocktail immunized rabbit P5672. The average percent neutralization and SDs from triplicate experiments are given. Orange, blue, and red curves show neutralization of these same viruses after absorption of the P5672 IgG with WITO (orange), C108 (blue), or CRF250 SOSIPs, respectively.

A

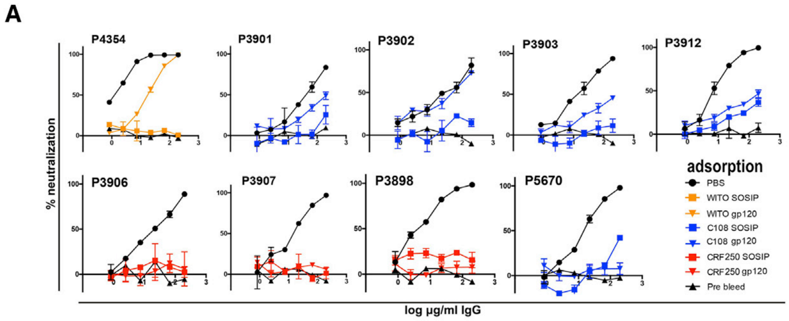

B

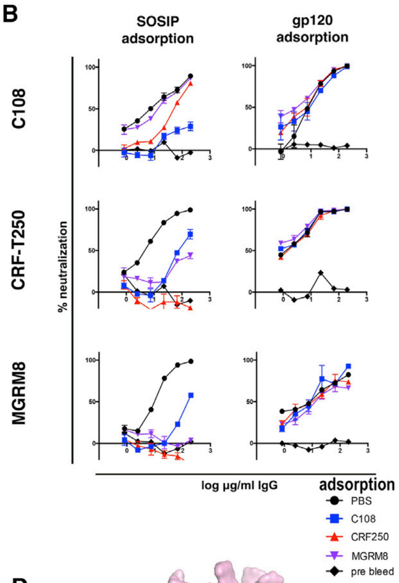

C

		P4354	P3901	P3902	P3903	P3912	P3906	P3907	P3898	P5670	P5672
wild type virus	C108		22.0	53.5	19.7	6.6				49.7	12.5
variants filling glycan holes	S130N (+N130)		49.4		95.7	7.8					16.2
	N188S (+N186)		47.9	73.9	32.2	6.6					13.6
	Y191S (+N189)		51.9			8.0					13.2
	K357T (+N355)		36.5	47.4	26.3	13.0				40.2	17.2
	S130N N188S		47.3	91.9	74.1	7.9					14.1
glyco-variants at apex	Kif		10.1	25.3	17.1	4.3				7.6	3.5
	N160S (ΔN160)		17.7	15.2	1.4	8.9				0.5	6.1
V2 basic strand C	K169T		61.8		76.3	17.4				[illegible]	19.9
	K169T K171E					9.9					21.7
wild type virus	CRF250						33.7	12.6	8.8		4.1
variants filling glycan holes	D130N K132T (+N130)						34.4	29.2	32.7		6.0
	N187S (+N185)						49.1	47.1			5.9
	R189S (+N187)						47.1				6.2
	K236T (+N234)						89.7	32.0	18.9		74.1
	I278T (+N276)							28.9	19.0		8.6
	D448N (+N448)						43.7	19.8	9.1		5.6
glyco-variants at apex	Kif						7.4	11.3	8.0	38.5	<1.23
	N156A (ΔN156)						2.4	7.9	10.4		1.0
	N160A (ΔN160)						71.9	23.4	15.1		9.3
V2 basic strand C	K169T						74.0	39.6	13.1		8.7
	K169T K171E						40.3	70.4	20.0		1.5
wild type virus	WITO	5.5									
variants filling glycan holes	H130N (+N130)	9.1									
	N188S (+N186)										
	G190S (+N188)	87.3									
	K236T (+N234)	3.8									
	K289N P291S (+N289)	2.8									
glyco-variants at apex	Kif	6.6								65.2	30.9
	N160A (ΔN160)	<0.4									
V2 basic strand C	I169K	7.3									
	K171T	5.3									
wild type virus	MGRM8										7.0
variants filling glycan holes	I130N (+N130)										11.4
	I236T (+N234)										89.5
glyco-variants at apex	Kif									77.2	2.5
Epitope targeted		V2 apex	V2 apex	V2 apex	V2 apex	?	234/276	V2 apex	V2 apex	V2 apex	234/276*

* CRF250 and MGRM8 activites

D

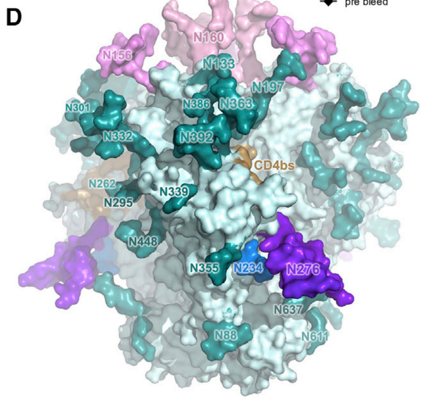

Figure 4. Specificities of Neutralizing Responses

(A and B) Trimer dependence of neutralizing abs for the 10 rabbits with high titer neutralizing responses in this experiment are shown. D368R autologous SOSIP or gp120 protein was used to adsorb IgG from bleed "3C" samples before neutralization in the TZMB-l assay. The plots in (A) are neutralization data for 9 rabbits that raised responses to the autologous virus only so the sequence-matched SOSIP or gp120 was used to adsorb IgG in each sample. The plots in (B) are neutralization data for the three viruses that were neutralized by SOSIP mixture immunized rabbit, P5672, after the second boost. Absorption of neutralization by WITO, C108, and CRF250-gp120 or SOSIP was assessed for each virus. Error bars represent SD between replicate data points.

(C) Envelope mutants of the viruses neutralized by rabbit immune IgG were generated as indicated in the left-hand column (HXB2 numbering). These include N160/N156 glycan knockouts, addition of PNG sites at predicted glycan holes, and mutation of the strand-C V2-apex bnAb core epitope of basic residues (169 and 171). In addition, wild-type viruses grown in the presence of kifunensine (Kif) were included. Neutralization IC_{50}s for these different pseudoviruses are given in μg/ml of rabbit IgG (identification number is given across the top). The limit of detection is 100 μg/mL. Titration curves are shown in Figure S4.

(D) BG505 trimer side view (PDB: 5XT3) showing the glycan hole targeted at 234/276 in some immunized rabbits (compare with C). Glycans are absent at positions 234 (blue) and 276 (purple) in CRF250 trimer and 234 in MGRM8. resulting in glycan holes.

was a difference in the cross-reactivity of antibody responses generated by the different SOSIP immunogens in gp120 ELISA (Figure S2A). C108 SOSIP trimer elicited the lowest levels of antibodies cross-reactive with CRF250, MGRM8 and WITO gp120s. All immunogens elicited SOSIP cross-reactive antibodies. The immunogens also elicited different levels of autologous V3 reactivity when assessed for by ELISA using sequence-matched V3 loop peptides (Figure S2A). C108 elicited relatively low V3 titers, consistent with its inability to elicit good tier 1 neutralizing responses or cross-reactive gp120 antibodies, which suggests that its V3 epitope remains fairly well concealed during immunization. V3 titers, like tier 1 neutralization, did not correlate with autologous neutralizing responses overall (Figure S2C).

Trimer Specificity of nAb Responses

To determine whether the neutralizing responses generated were SOSIP trimer specific, we carried out IgG adsorption assays by pre-incubating IgG purified at the B3C time point with excess sequence-matched D368R trimer or gp120 protein (Figure 4A). As expected, sequence-matched SOSIP trimers adsorbed neutralization activity from all samples (Figure 4A). With

respect to sequence-matched monomeric gp120 adsorption, we found that the neutralizing activity was completely adsorbed from the CRF250-immunized group in all three neutralizing rabbits, indicating no trimer-specific neutralizing IgG responses were elicited. The neutralizing samples from WITO, and 3 of 4 C108 immunized rabbits, showed only partial adsorption by sequence-matched gp120, suggesting some preference for a trimeric quaternary epitope. Only one C108-immunized animal (P3902) showed no adsorption by gp120 and presumed full dependence on a trimeric quaternary structure for neutralizing activity (Figure 4A).

We performed SOSIP/gp120 adsorption assays on the serum IgG from rabbit P5672. Sequence-matched SOSIP trimers adsorbed the C108, CRF250, and MGRM8 neutralizing activities in this sample as expected, but there was also some cross-strain adsorption of neutralizing activity. CRF250 completely adsorbed the neutralizing activity against MGRM8 virus, suggesting the presence of MGRM8/CRF250 cross-nAbs in this rabbit. None of the immunogen-matched gp120s adsorbed autologous neutralization against any virus, suggesting a dependence of all neutralizing activities on trimeric Env in this rabbit (Figure 4B).

Autologous Neutralizing Responses Predominantly Focus on the Lysine-Rich Region that Forms the Core Epitope for V2-Apex bnAbs

We hypothesized that neutralizing responses were targeting glycan holes on the neutralizing surfaces of the closed trimer immunogens, similar to observations with other SOSIP immunogens (Klasse et al., 2016; McCoy et al., 2016). Therefore, we generated a panel of virus variants in which PNG sites were added at the positions of the putative glycan holes (Figure S3B). We then assayed the effects on the neutralizing activity of IgG samples (Figures 4C and S4A). PNG sites introduced at the V2 apex at residue 130 or within the V2′ region (183–191) reduced the neutralizing activity in seven of the ten neutralizing rabbit IgG samples. These included the only animal that developed neutralizing activity against WITO (P4354), four of the six C108 neutralizing responses (P3901, 3902, 3903, and 5670), and two of the four CRF250 neutralizing responses (3907 and P3898). A similar sensitivity to filling in the glycan holes is seen for the iP versions of prototype V2-apex bnAbs (Figure 1A). This result demonstrates that SOSIP trimers lacking glycans at 130 and within the V2′ region can immunofocus autologous neutralizing responses onto epitopes overlapping those bound by prototype bnAb iPs. To further explore the specificity of the predominant rabbit responses, viruses were made with substitutions in the strand-C basic lysine patch (K169/K171) and with substitutions to remove the N160/N156 glycans, all critical for prototype V2-apex bnAb neutralization (Andrabi et al., 2015). Remarkably, all four C108-elicited neutralization responses, which mapped to the V2 apex, also relied on the strand-C basic patch residues for activity (Figures 4C and S4A). In general, neutralizing responses were unaffected or improved in virus variants lacking the N160 or N156 glycans or against viruses grown in the presence of kifunensine (kif) (Figures 4C and S4A).

A minority of neutralizing responses elicited in this study targeted a second glycan hole common to only the CRF250 (HXB2 234/276) and MGRM8 (HXB2 234) immunogens (Figure S3B). These include the CRF250 activity elicited in animal P3906 and both the CRF250 and MGRM8 responses elicited in rabbit P5672 immunized using the SOSIP mixture (Figures 4C and S4A). Overall, the neutralizing responses induced in this study were directed to both trimer-specific epitopes and those found on monomeric gp120. The responses predominantly targeted glycan holes around the apex and the lysine residues that form the core epitope for V2-apex bnAbs.

Neutralizing mAbs Isolated from C108- and CRF250-Trimer-Immunized Rabbits Partially Recapitulate the Corresponding Serum Specificity

To further identify and characterize the fine specificities of the trimer-induced neutralizing responses, we sorted antigen-specific B cells from PBMCs using immunogen-matched trimer probes. The heavy-chain (V_H) and light-chain (V_L) transcripts from antigen-specific B cells were recovered by single-cell PCR amplification as previously described (McCoy et al., 2016). Only six nAbs were identified from hundreds of antigen-specific B cells derived from all ten neutralizing rabbit PBMC samples, showing that the overwhelming majority of specific antibodies are directed to non-neutralizing epitopes. The neutralizing mAbs include three from one C108-immunized rabbit (P3903; mAbs 3903-1, 3903-2, and 3903-3) and three from two CRF250-immunized rabbits (one from P3907 [mAb 3907-1] and two from P3898 [mAbs 3898-1 and 3898-2]). Antibody germline V, D, and J genes were assigned using the international immunogenetics information system (IMGT) database (Lefranc et al., 1999) and are shown in Figure 5A. The antibodies had CDRH3s between 10 and 17 residues in length, while complementarity-determining region 3 of the light chains (CDRL3s) were between 10 and 15 residues, which is longer than those typically seen in human antibodies. Most mAbs possessed a YD motif at the center of their CDRL3s (at the V-J gene junction), reminiscent of the YYD motif in CAP256.09 and PG9 CDRH3s that are associated with binding to the strand-C basic residues (Figures 5A and S5A). One mAb (3907-1) had a YDDY motif centered on the middle of its 16-amino-acid CDRH3. Two of the three C108 mAbs from animal P3903 (1 and 2) appear to be members of the same lineage despite differing assignments of their light chain (LC) germline V-genes (Figure S5A).

Epitope specificities of the mAbs were mapped in the same way as the polyclonal rabbit serum responses shown in Figure 4C. Mostly, mAbs recapitulated the properties of the sera from the source rabbit, with some variations with regards to the effect of the Env glycan variants. Activities of all three C108-specific neutralizing mAbs relied on the strand-C basic residues K169 and K171, as seen for all V2-apex bnAb prototypes. PNG sites introduced to fill glycan holes at 130 or within the V2′ region could reduce their activity, and the effect was strongest for N130, as was observed for iP versions of prototype human bnAbs (Figures 5B and S4B). The activity of the CRF250-elicited mAbs also showed sensitivity to the strand-C lysines but were variably tolerant of glycans at N130 and within the V2′ (Figures 5B and S4B). No mAb could neutralize the heterologous isolates assayed.

Individual mAbs did not necessarily reflect sera in terms of the effects of kif on virus neutralization. Three mAbs showed

A

nAB	chain	V-GENE/allele	identity %	D-GENE/allele	J-GENE/allele	identity %	CDR lengths	AA JUNCTION (CDR3)	EC50(ug/ml)
3903-1	H	Orycun IGHV1S44*01 F	92.0	IGHD8-1*01 F	IGHJ3*01 F	69.8	8.7.10	CARYAISSADLW	0.56
	K	Orycun IGKV1S23*01 F	86.7		IGKJ1-2*01 F	91.4	8.3.10	CGAGYDGNIYVF	
3903-2	H	Orycun IGHV1S44*01 F	91.7	IGHD8-1*01 F	IGHJ3*01 F	69.8	8.7.10	CTRYAISSADLW	0.22
	K	Orycun IGKV1S64*01 [F]	89.8		IGKJ1-2*01 F	91.4	8.3.10	CGGGYDGNIYVF	
3903-3	H	Orycun IGHV1S45*01 F	74.9	IGHD4-1*01 F	IGHJ4*01 F	83.3	8.7.13	CAGSSDGDWNYFNLW	0.6
	K	Orycun IGKV1S61*01 [F]	91.8		IGKJ1-2*01 F	97.2	8.3.13	CLGEYDCSISDCNVF	
3907-1	H	Orycun IGHV1S45*01 F	76.7	IGHD2-1*01 F	IGHJ2*01 F	88.0	9.8.16	CARENYDDYGDSHAFDPW	0.08
	K	Orycun IGKV1S10*01 F	91.4		IGKJ1-2*01 F	95.0	6.3.15	CQSNDGISSSSYGPNAF	
3898-1	H	Orycun IGHV1S45*01 F	90.7	IGHD6-1*01 F	IGHJ4*01 F	81.3	9.9.13	CARDDTADDDFYDLW	0.28
	K	Orycun IGKV1S15*01 F	89.1		IGKJ1-2*01 F	95.0	8.3.12	CLGSYDCSVDCNAF	
3898-2	H	Orycun IGHV1S69*01 F	90.2	IGHD3-3*01 F	IGHJ3*01 F	71.7	8.7.17	CARGHYFSDGSGITTLVIW	0.34
	K	Orycun IGKV1S54*01 [F]	90.9		IGKJ1-2*01 F	97.4	8.3.11	CLGSYDDDGDNTF	

B

		3903 serum	3903-1	3903-2	3903-3
wild type virus	C108	19.7	0.07	0.1	0.59
variants filling holes	S130N (+N130)	95.7	>5	>5	>5
	N188S (+N186)	32.2	2.9	0.4	>5
	S130N N188S	74.1	>5	>5	>5
glyco-variants at apex	Kif	17.1	>5	>5	0.11
	N160S (ΔN160)	1.4	<0.02	<0.02	0.05
V2 basic strand C	K169T	76.3	0.7	0.17	0.73
	K169T K171E	>100	>5	>5	>5

C

		3907 serum	3907-1	3898 serum	3898-1	3898-2
wild type virus	CRF250	12.6	0.05	8.8	<0.02	<0.02
variants filling holes	D130N K132T (+N130)	29.2	0.13	32.7	>5	1.3
	N187S (+N185)	47.1	>5	>100	>5	3.8
	R189S (+N187)	>100	>5	>100	>5	>5
glyco-variants at apex	N156A (ΔN156)	7.9	4.7	10.4	>5	1.4
	N160A (ΔN160)	23.4	0.14	15.1	<0.02	0.04
	Kif	11.3	>5	8.0	<0.02	0.02
V2 basic strand C	K169T	39.6	0.62	13.1	0.03	0.08
	K169T K171E	70.4	>5	20.0	1.2	0.4

Figure 5. Neutralizing Monoclonal Rabbit Antibodies Derived from Three Different Immunized Animals

(A) Heavy and light (kappa) chains including genetic identity to their most similar *O. cuniculus* germline genes (from the IMGT database), CDR lengths, and CDR3 sequences. YD motifs in the heavy CDRH3 or kappa CDRL3 loops are highlighted.

(B) Neutralization IC_{50} values of three C108-specific mAbs (in micrograms per milliliter) are given to the right of values for the parent rabbit immune serum IgG IC_{50} values for a variety of C108 variants (left column). 3903-1 and 3903-2 are members of a clonal lineage (Figure S5A).

(C) Neutralization IC_{50} values of CRF250-specific mAbs derived from two different animals. As in (B), parent immune serum IgG IC_{50} values are given to the left of the values for the mAbs against the virus variants indicated in the left column.

enhanced or unaltered neutralization of virus grown in kif-treated cells. In contrast, a complete loss of the neutralizing activity was observed for three other mAbs, a property typical of most V2-apex bnAb prototypes (Andrabi et al., 2015; Doores and Burton, 2010).

Elimination of N160 and N156 sites had varying effects on autologous neutralization by mAbs. N160 elimination improved neutralization activity for all C108-specific mAbs, while eliminating the N156 glycan site rendered C108 virus non-functional, precluding investigation of neutralization. Elimination of N160 had a minimal effect on CRF250 mAb activity. One CRF250 V2-apex-specific mAb required a glycan at N156 for activity, and the two others showed reduced neutralization without this glycan (Figures 5C and S4B), in contrast to the polyclonal neutralizing response of the source rabbit, which was minimally affected.

In all, the six V2-apex-reactive neutralizing mAbs isolated in this study only partially reproduced neutralizing activities present in the source animals. However, they illustrate that a diversity of autologous responses to the V2 apex can be elicited, and some of these recapitulate properties of iP versions of prototype V2-apex bnAbs, including a requirement for basic residues at 169/171 and a sensitivity to glycans at 130 and within the V2′ region. Some mAbs also require N160 and/or N156 glycans for neutralization.

DISCUSSION

HIV vaccine strategies that target specific naive B cells followed by boosts designed to shepherd primed responses toward bnAbs have recently attracted much attention (Burton, 2017; Haynes and Mascola, 2017; Klein et al., 2013; Xiao et al., 2009). For the quaternary V2-apex epitope targets, native Env trimers are likely to be the most appropriate immunogens, and thus, it is key to identify trimers that bind to naive B cell receptors (BCRs) that possess the potential to evolve into bnAb lineages. In vitro binding to iP versions of bnAbs is encouraging, but the true test of value comes in whether the trimers can elicit the appropriate antibodies in vivo. Here, we investigated a range of V2-apex bnAb-iP-binding trimers as immunogens in a rabbit model.

First, we showed that a glycan hole (Crooks et al., 2015; Klasse et al., 2016; McCoy et al., 2016) at the trimer apex, resulting from the absence of PNG sites at positions 130 and within the V2′, was strongly associated with binding of iP versions of V2-apex bnAbs. Subsequently, we showed that SOSIP trimers with this glycan hole successfully elicited apex-focused neutralizing responses in rabbits. While induction of V1V2-specific responses has been reported previously in several species (Liao et al., 2013; Zolla-Pazner et al., 2016), none described vaccine elicitation of V2-focused neutralizing responses.

The SOSIP immunogens described here differed in their ability to reproducibly elicit apex-neutralizing responses. Trimers C108, CRF250, WITO, and MGRM8 elicited neutralization that could be mapped to the apex in 3 out of 4, 2 out of 4, 1 out of 4, and 0 out of 4 animals, respectively. With a caveat that the group sizes are small, there are several possible reasons these immunogens might elicit neutralizing responses to this epitope with differing consistency: (1) The presence of a glycan hole may be important, as the only immunogen that failed to elicit V2-apex-directed neutralizing responses was MGRM8 SOSIP trimer, which has the least extensive glycan hole. This trimer lacks N130 but it does possess a PNG site within the V2′ region. The V2-apex glycan hole in C108, CRF250, and WITO immunogens should

be similar in size, as these isolates possess identical PNG sites in this region of the trimer. Other factors may therefore contribute to differences in immunogenicity here. (2) Differing immunogen stability. The most successful trimer, C108, showed the highest proportion of trimers in a tightly closed native conformation by negative-stain EM and was the most thermostable. In addition, this immunogen had the lowest reactivity to non-neutralizing human mAbs and elicited the lowest levels of tier 1 neutralizing, V3-binding, and gp120 cross-reactive antibodies in rabbits. These data suggest that in addition to being a tightly packed trimer with minimal exposure of non-neutralizing epitopes, this immunogen is stable enough to maintain conformation throughout the immunization process, potentially contributing to its ability to focus responses onto neutralizing epitopes like the apex glycan hole. In general, trimer thermostability was associated with an improved ability to reproducibly elicit autologous neutralization, as has been demonstrated previously (Feng et al., 2016). (3) Differing V2-apex iP reactivity. The most effective immunogens, C108 and CRF250, bind all of the PG9, CH01, and CAP256 prototype iPs, while WITO and MGRM8 are neutralized by only two of these antibodies. Further, WITO and MGRM8 SOSIPs showed the poorest binding to iP/UCA antibodies by BLI, suggesting there are potentially fewer ways for them to trigger naive BCRs. (4) Differing strand-C basic residue sequences. The V2-apex strand-C patch (HXB2 168–171) makes essential contact with prototype V2-apex bnAbs in the form of strong polar interactions involving basic side chains on Env. Notably, one of the trimers that induced less reproducible neutralization, WITO, has an isoleucine residue at the 169 position instead of the more common lysine, which may negatively impact the ability to induce appropriate V2-targeted Abs. Indeed, the limited neutralization coverage of the CAP256 V2-apex bnAbs against the clade B viruses has been associated with the lack of a positively charged residue at position 169 (Doria-Rose et al., 2014, 2015). Therefore, the chemical nature of strand-C residues in the V2-apex prime and boost immunogens may need to be tightly regulated to favor responses along bnAb developmental pathways.

In addition to comparing four iP targeting SOSIPs as immunogens individually, 2 out of 4 rabbits immunized with the SOSIP mixture developed high-titer nAbs to the C108 virus, and one of these (P5672) neutralized viruses corresponding to two of the other immunogens (CRF250 and MGRM8). The mixture was uniquely able to elicit potent neutralizing responses to MGRM8, while 4 out of 4 animals immunized with MGRM8 alone failed to do the same. CRF250 neutralizing activity in P5672 was trimer dependent, whereas all responses elicited using CRF250 immunogen alone were gp120 specific, suggesting a modulation of the neutralizing response to CRF250 in the context of the SOSIP mixture. Animal P5672 also uniquely elicited nAbs to five heterologous tier 2 viruses, and it is of interest to gain a better understanding of how this was accomplished.

The range of trimer dependencies in C108- and WITO-immunized animals is interesting given the dominant epitope targeted was mapped to the V2 apex. This epitope is generally trimer dependent for prototype bnAbs. While rare, some strains of HIV, including C108, can bind to PG9 and PG16 bnAbs as monomeric gp120s (Figure S1C). This suggests it may be possible to elicit nAbs to this epitope with a range of trimer dependencies for some strains. Alternatively, neutralizing responses may target a V2-apex epitope that does not completely overlap with prototype bnAbs and may not be trimer dependent.

We isolated neutralizing mAbs to evaluate whether they possessed lineage properties that show promise for eventual affinity maturation into bnAbs. Like human V2-apex prototype bnAbs, all neutralizing rabbit mAbs recognize the V2 basic region of strand-C lysines (K169/K171) to some degree. Because CDRL3s lengths are longer in the rabbit antibody repertoire than in the human (Lavinder et al., 2014), contact through this complementarity-determining region (CDR) is a distinct possibility. Encouragingly, we found that three out of six mAbs showed dependence on glycans similar to human V2-apex bnAbs (Andrabi et al., 2015), and some mAbs were able to tolerate glycans introduced to fill the V2-apex glycan hole, even at position 130. These features are promising for further affinity maturation toward broad HIV neutralization.

In summary, this study describes reproducible vaccine-elicited tier 2 autologous nAb responses directed toward an epitope that overlaps that of prototype V2-apex-directed bnAbs in an outbred animal model, and it provides a starting point from which to test boosting strategies designed to broaden these responses to generate V2-apex bnAbs capable of protecting against global HIV isolates.

EXPERIMENTAL PROCEDURES

Antibodies and Virus Envelopes

Mature, iP, and UCA antibody sequences used in this study are described in Andrabi et al. (2015) and Doria-Rose et al. (2014). Virus envelopes from reference strains previously described (Seaman et al., 2010) were obtained from NIH AIDS Research and Reference Reagent Program (ARRR). The sequence of MGRM_AG_002_8 (subtype AG) was obtained from Monogram Biosciences and cloned in to the pSVIII vector as described elsewhere (Helseth et al., 1990). Amino acid point mutations in HIV envelopes were made using a QuikChange site-directed mutagenesis kit (Stratagene) according to the manufacturer's instructions. All mutations were verified by DNA sequence analysis (Eton Bioscience).

Virus Production and Neutralization Assay

Single-round infectious HIV-1 Env pseudoviruses were made as described previously (Seaman et al., 2010). Briefly, plasmids encoding Env were cotransfected with an Env-deficient backbone plasmid (pSG3ΔENV) using Fugene 6 (Promega). For kif-grown viruses, 25 μM kif was added to 293T cells on the day of transfection. Cell media were harvested 48 hr post-transfection and stored at −80°C. Neutralization of pseudoviruses by IgG samples was measured on TZM-bl target cells, as described previously (Seaman et al., 2010). Neutralizing rabbit serum IgG or mAbs were reported as the antibody concentration that resulted in 50% virus neutralization (IC_{50}) in micrograms IgG per milliliter after fitting the curve of log[antibody] versus percent neutralization in Prism. For neutralization absorption assays, 0.1 mg/mL D368R gp120 or SOSIP was incubated with 200 μg/mL IgG for 1 hr at 37°C before adding virus and proceeding as above.

Generation of iP-Binding Soluble Native Trimers

HIV-1 Env amino acid sequences (GenBank: JN944660 [C108.c03]; GenBank: EU13189 [CRF-T250-4]; GenBank: AY835451 [WITO.4130]; GenBank: MF510462 [MGRM-002-8]) were used to generate codon-optimized SOSIP.664 gp140 genes (Geneart, Life Technologies) for expression of soluble native trimers as described in Sanders et al. (2013). Tissue plasminogen activator (TPA) leader sequences were used and genes were cloned into phCMV vectors (Genlantis) for transient transfection in 293F cells. Secreted trimers

were purified from cell supernatants after 5 days using PGT145 bnAb antibody affinity columns as described previously (Pugach et al., 2015). D368R versions of these proteins and sequence-matched gp120s were expressed in the same way and purified using *Gallanthus nivalis* lectin (GNL) (Vector Labs). Affinity-purified proteins were purified by size exclusion chromatography using a Superdex 200 10-300 GL column (GE Healthcare) in PBS.

Trimer Characterization

N-glycan site occupancy of trimers was performed as described in Behrens et al. (2016). Negative-stain EM, DSC, and BLI/Octet were performed as described elsewhere (Guenaga et al., 2015).

Ethics Statement

This study was approved and carried out in accordance with protocols provided to the Institutional Animal Care and Use Committee (IACUC) at The Scripps Research Institute (TSRI; La Jolla, CA) under approval number 14-0002. The rabbits were kept, immunized, and bled at TSRI in compliance with the Animal Welfare Act and other federal statutes and regulations relating to animals and in adherence to the *Guide for the Care and Use of Laboratory Animals* (National Research Council, 1996).

Immunization and Sampling

20 female 12-week-old New Zealand white rabbits were primed and boosted with 50 μg protein and 100 U Iscomatrix in 600 μL PBS (300 μL IM injection into each hind leg). Blood was drawn from the marginal ear vein into EDTA or untreated blood collection tubes. All procedures were performed in anesthetized animals. EDTA plasmas were diluted three times with PBS and PBMCs purified on a Lymphoprep (STEMCELL Technology) density gradient. PBMCs were cryopreserved in FBS plus 10% DMSO.

Purification of Polyclonal IgG from Serum

Clotted, filtered serum samples were mixed with protein A/G and incubated overnight at 4°C with agitation. Beads were washed with PBS and eluted using 0.1 M citric acid (pH 3.0) and neutralized with 1 M Tris (pH 9.0). Purified IgG was buffer exchanged into PBS and stored at 200 μg/mL at 4°C for neutralization and ELISAs.

ELISA

SOSIP ELISA reagents were randomly biotinylated using a 2:1 molar ratio of biotin reagent to trimer using the EZ-link-NHS-PEG$_4$-Biotin kit (Thermo Fisher Scientific, 21324) MaxiSorp plates (Thermo Fisher Scientific) were coated overnight at 4°C with 2 μg/mL gp120 protein or 2 μg/mL streptavidin (Thermo Fisher Scientific). Plates were blocked for 1 hr with 3% BSA and washed three times with 0.05% Tween 20-PBS (PBS-T) (pH 7.4). Streptavidin-coated plates were incubated with randomly biotinylated SOSIPs in 1% BSA plus PBS-T for 2 hr and washed three times with PBS-T. Purified rabbit serum IgG or human mAbs were added at a maximum concentration of 200 μg and 10 μg/mL, respectively, serially diluted 1:6 in 1% BSA plus PBS-T, and incubated at room temperature (RT) for 1 hr. Plates were washed three times with PBS-T. Alkaline-phosphatase-conjugated goat anti-human or anti-rabbit IgG Fc secondary antibody (Jackson ImmunoResearch Laboratories) was diluted 1:500 in 1% BSA PBS-T and added to plates for 1 hr at RT. Plates were washed three times with PBS-T and incubated with phosphatase (Sigma) for 60 min and the absorbance at 405 nm recorded. The bnAb reactivity with biotinylated SOSIPs proved that these reagents contained native epitopes. Direct coating of gp120 on plates showed similar results but better dynamic range than gp120 capture using an anti-C5 antibody.

Rabbit Monoclonal nAbs

Fluorescence-activated cell sorting of cryopreserved PBMCs was performed as described in McCoy et al. (2016). Briefly, IgM−, IgG+, antigen-specific B cells were sorted using randomly biotinylated (Thermo Fisher Scientific, 20217), PGT145-purified SOSIPs (sequence matched to the autologous neutralizing activity in the animal from which the B cells were obtained) as streptavidin-allophycocyanin-bound tetramers (Thermo Fisher Scientific, SA1005). Single cells were sorted into 96-well plates for PCR amplification of heavy and kappa chains using previously described primers and PCR conditions (McCoy et al., 2016). Rabbit antibody variable regions were cloned into an expression plasmid adapted from the pFUSE-rIgG-Fc and pFUSE2-CLIg-rK2 vectors (InvivoGen) under ampicillin selection.

Statistical Analysis

Correlations in data reported in Figures 2 and S2C were assessed in Prism using a two-tailed Spearman's rank correlation coefficient. p values less than 0.05 were considered significant. Heterologous tier 2 virus neutralization titration curves reported in Figure 3C are the average percent neutralizations from three replicate experiments with SDs reported as bars above and below the data points.

DATA AND SOFTWARE AVAILABILITY

The accession number for the envelope nucleotide sequence from called AG HIV isolate MGRM_002_8 (obtained from Monogram and previously unpublished) is GenBank: MF510462. The accession numbers for the monoclonal rabbit antibody sequences are GenBank: MF510450 and MF510451 (3903-1); MF510452 and MF510453 (3903-2); MF510454 and MF510455 (3903-3); MF510456 and MF510457 (3907-1); MF510458 and MF510459 (3907-1); and MF510460 and MF510461 (3898-1)..

SUPPLEMENTAL INFORMATION

Supplemental Information includes five figures and can be found with this article online at https://doi.org/10.1016/j.celrep.2017.09.024.

AUTHOR CONTRIBUTIONS

J.E.V., R.A., and L.E.M. designed the experiments. J.E.V., R.A., L.E.M., N.d.V., R.P.F., T.M., C.-Y.S., D.S., S.N.K., F.G., and L.K.P. performed the experiments. R.T.W., A.B.W., M.C., and I.A.W. contributed critical reagents. J.E.V., R.A., and D.R.B. analyzed the data and wrote the paper.

ACKNOWLEDGMENTS

We thank Monogram for viral envelope sequences. We also thank Justin Orman and the staff at the Department of Animal Resources at The Scripps Research Institute for animal housing and care, as well as performing immunizations and sample collections. We thank Michel Nussenzweig, James Robinson, John Mascola, Miroslaw Gorny, and Susan Zolla-Pazner for providing antibodies directly or through the IAVI Neutralizing Antibody Consortium (Scripps) or NIH AIDS Research and Reference Reagent Program (ARRRP). We thank Sergey Menis and Sebastian Raemisch in the laboratory of William Schief at TSRI for a BG505 structure with N-linked glycans modeled in. We thank Christina Corbaci for her help in the preparation of figures. This work was supported by the International AIDS Vaccine Initiative (IAVI) through the Neutralizing Antibody Consortium (NAC) (IAVI SFP 2120/2121 to D.R.B., R.W., A.B.W., and I.A.W.), the National Institute of Allergy and Infectious Diseases (Center for HIV/AIDS Vaccine Immunology and Immunogen Discovery grant UM1AI100663 to D.R.B., A.B.W., I.A.W., and M.C.), by the Ragon Institute of MGH, MIT, and Harvard (MGH/RAGON SFP to D.R.B.), and through USAID (PATH/USAID SFP to D.R.B.). L.E.M. receives support from an FP7 Marie Curie International Outgoing Fellowship for Career Development (number 623038). This study was made possible by the generous support of the Bill and Melinda Gates Foundation Collaboration for AIDS Vaccine Discovery (CAVD #OPP1084519).

Received: March 15, 2017
Revised: June 17, 2017
Accepted: September 6, 2017
Published: October 3, 2017

REFERENCES

Andrabi, R., Voss, J.E., Liang, C.H., Briney, B., McCoy, L.E., Wu, C.Y., Wong, C.H., Poignard, P., and Burton, D.R. (2015). Identification of common features

in prototype broadly neutralizing antibodies to HIV envelope V2 apex to facilitate vaccine design. Immunity *43*, 959–973.

Behrens, A.J., Vasiljevic, S., Pritchard, L.K., Harvey, D.J., Andev, R.S., Krumm, S.A., Struwe, W.B., Cupo, A., Kumar, A., Zitzmann, N., et al. (2016). Composition and antigenic effects of individual glycan sites of a trimeric HIV-1 envelope glycoprotein. Cell Rep. *14*, 2695–2706.

Bhiman, J.N., Anthony, C., Doria-Rose, N.A., Karimanzira, O., Schramm, C.A., Khoza, T., Kitchin, D., Botha, G., Gorman, J., Garrett, N.J., et al. (2015). Viral variants that initiate and drive maturation of V1V2-directed HIV-1 broadly neutralizing antibodies. Nat. Med. *21*, 1332–1336.

Bonomelli, C., Doores, K.J., Dunlop, D.C., Thaney, V., Dwek, R.A., Burton, D.R., Crispin, M., and Scanlan, C.N. (2011). The glycan shield of HIV is predominantly oligomannose independently of production system or viral clade. PLoS ONE *6*, e23521.

Bonsignori, M., Hwang, K.K., Chen, X., Tsao, C.Y., Morris, L., Gray, E., Marshall, D.J., Crump, J.A., Kapiga, S.H., Sam, N.E., et al. (2011). Analysis of a clonal lineage of HIV-1 envelope V2/V3 conformational epitope-specific broadly neutralizing antibodies and their inferred unmutated common ancestors. J. Virol. *85*, 9998–10009.

Burton, D.R. (2002). Antibodies, viruses and vaccines. Nat. Rev. Immunol. *2*, 706–713.

Burton, D.R. (2017). What are the most powerful immunogen design vaccine strategies? Reverse vaccinology 2.0 shows great promise. Cold Spring Harb. Perspect. Biol. *3*, a030262.

Burton, D.R., and Hangartner, L. (2016). Broadly neutralizing antibodies to HIV and their role in vaccine design. Annu. Rev. Immunol. *34*, 635–659.

Crooks, E.T., Tong, T., Chakrabarti, B., Narayan, K., Georgiev, I.S., Menis, S., Huang, X., Kulp, D., Osawa, K., Muranaka, J., et al. (2015). Vaccine-elicited tier 2 HIV-1 neutralizing antibodies bind to quaternary epitopes involving glycan-deficient patches proximal to the CD4 binding site. PLoS Pathog. *11*, e1004932.

Doores, K.J., and Burton, D.R. (2010). Variable loop glycan dependency of the broad and potent HIV-1-neutralizing antibodies PG9 and PG16. J. Virol. *84*, 10510–10521.

Doria-Rose, N.A., Schramm, C.A., Gorman, J., Moore, P.L., Bhiman, J.N., DeKosky, B.J., Ernandes, M.J., Georgiev, I.S., Kim, H.J., Pancera, M., et al.; NISC Comparative Sequencing Program (2014). Developmental pathway for potent V1V2-directed HIV-neutralizing antibodies. Nature *509*, 55–62.

Doria-Rose, N.A., Bhiman, J.N., Roark, R.S., Schramm, C.A., Gorman, J., Chuang, G.Y., Pancera, M., Cale, E.M., Ernandes, M.J., Louder, M.K., et al. (2015). New member of the V1V2-directed CAP256-VRC26 lineage that shows increased breadth and exceptional potency. J. Virol. *90*, 76–91.

Feng, Y., Tran, K., Bale, S., Kumar, S., Guenaga, J., Wilson, R., de Val, N., Arendt, H., DeStefano, J., Ward, A.B., and Wyatt, R.T. (2016). Thermostability of well-ordered HIV spikes correlates with the elicitation of autologous tier 2 neutralizing antibodies. PLoS Pathog. *12*, e1005767.

Georgiev, I.S., Doria-Rose, N.A., Zhou, T., Kwon, Y.D., Staupe, R.P., Moquin, S., Chuang, G.Y., Louder, M.K., Schmidt, S.D., Altae-Tran, H.R., et al. (2013). Delineating antibody recognition in polyclonal sera from patterns of HIV-1 isolate neutralization. Science *340*, 751–756.

Gorman, J., Soto, C., Yang, M.M., Davenport, T.M., Guttman, M., Bailer, R.T., Chambers, M., Chuang, G.Y., DeKosky, B.J., Doria-Rose, N.A., et al.; NISC Comparative Sequencing Program (2016). Structures of HIV-1 Env V1V2 with broadly neutralizing antibodies reveal commonalities that enable vaccine design. Nat. Struct. Mol. Biol. *23*, 81–90.

Gristick, H.B., von Boehmer, L., West, A.P., Jr., Schamber, M., Gazumyan, A., Golijanin, J., Seaman, M.S., Fätkenheuer, G., Klein, F., Nussenzweig, M.C., and Bjorkman, P.J. (2016). Natively glycosylated HIV-1 Env structure reveals new mode for antibody recognition of the CD4-binding site. Nat. Struct. Mol. Biol. *23*, 906–915.

Guenaga, J., de Val, N., Tran, K., Feng, Y., Satchwell, K., Ward, A.B., and Wyatt, R.T. (2015). Well-ordered trimeric HIV-1 subtype B and C soluble spike mimetics generated by negative selection display native-like properties. PLoS Pathog. *11*, e1004570.

Haynes, B.F., and Mascola, J.R. (2017). The quest for an antibody-based HIV vaccine. Immunol. Rev. *275*, 5–10.

Helseth, E., Kowalski, M., Gabuzda, D., Olshevsky, U., Haseltine, W., and Sodroski, J. (1990). Rapid complementation assays measuring replicative potential of human immunodeficiency virus type 1 envelope glycoprotein mutants. J. Virol. *64*, 2416–2420.

Julien, J.P., Lee, J.H., Cupo, A., Murin, C.D., Derking, R., Hoffenberg, S., Caulfield, M.J., King, C.R., Marozsan, A.J., Klasse, P.J., et al. (2013). Asymmetric recognition of the HIV-1 trimer by broadly neutralizing antibody PG9. Proc. Natl. Acad. Sci. USA *110*, 4351–4356.

Klasse, P.J., LaBranche, C.C., Ketas, T.J., Ozorowski, G., Cupo, A., Pugach, P., Ringe, R.P., Golabek, M., van Gils, M.J., Guttman, M., et al. (2016). Sequential and simultaneous immunization of rabbits with HIV-1 envelope glycoprotein SOSIP.664 trimers from clades A, B and C. PLoS Pathog. *12*, e1005864.

Klein, F., Mouquet, H., Dosenovic, P., Scheid, J.F., Scharf, L., and Nussenzweig, M.C. (2013). Antibodies in HIV-1 vaccine development and therapy. Science *341*, 1199–1204.

Landais, E., Huang, X., Havenar-Daughton, C., Murrell, B., Price, M.A., Wickramasinghe, L., Ramos, A., Bian, C.B., Simek, M., Allen, S., et al. (2016). Broadly neutralizing antibody responses in a large longitudinal sub-saharan HIV primary infection cohort. PLoS Pathog. *12*, e1005369.

Lavinder, J.J., Hoi, K.H., Reddy, S.T., Wine, Y., and Georgiou, G. (2014). Systematic characterization and comparative analysis of the rabbit immunoglobulin repertoire. PLoS ONE *9*, e101322.

Lee, J.H., Ozorowski, G., and Ward, A.B. (2016). Cryo-EM structure of a native, fully glycosylated, cleaved HIV-1 envelope trimer. Science *351*, 1043–1048.

Lefranc, M.P., Giudicelli, V., Ginestoux, C., Bodmer, J., Müller, W., Bontrop, R., Lemaitre, M., Malik, A., Barbié, V., and Chaume, D. (1999). IMGT, the international ImMunoGeneTics database. Nucleic Acids Res. *27*, 209–212.

Liao, H.X., Bonsignori, M., Alam, S.M., McLellan, J.S., Tomaras, G.D., Moody, M.A., Kozink, D.M., Hwang, K.K., Chen, X., Tsao, C.Y., et al. (2013). Vaccine induction of antibodies against a structurally heterogeneous site of immune pressure within HIV-1 envelope protein variable regions 1 and 2. Immunity *38*, 176–186.

McCoy, L.E., and Burton, D.R. (2017). Identification and specificity of broadly neutralizing antibodies against HIV. Immunol. Rev. *275*, 11–20.

McCoy, L.E., van Gils, M.J., Ozorowski, G., Messmer, T., Briney, B., Voss, J.E., Kulp, D.W., Macauley, M.S., Sok, D., Pauthner, M., et al. (2016). Holes in the glycan shield of the native HIV envelope are a target of trimer-elicited neutralizing antibodies. Cell Rep. *16*, 2327–2338.

McLellan, J.S., Pancera, M., Carrico, C., Gorman, J., Julien, J.P., Khayat, R., Louder, R., Pejchal, R., Sastry, M., Dai, K., et al. (2011). Structure of HIV-1 gp120 V1/V2 domain with broadly neutralizing antibody PG9. Nature *480*, 336–343.

Moldt, B., Rakasz, E.G., Schultz, N., Chan-Hui, P.Y., Swiderek, K., Weisgrau, K.L., Piaskowski, S.M., Bergman, Z., Watkins, D.I., Poignard, P., and Burton, D.R. (2012). Highly potent HIV-specific antibody neutralization in vitro translates into effective protection against mucosal SHIV challenge in vivo. Proc. Natl. Acad. Sci. USA *109*, 18921–18925.

Moore, P.L., Gray, E.S., Wibmer, C.K., Bhiman, J.N., Nonyane, M., Sheward, D.J., Hermanus, T., Bajimaya, S., Tumba, N.L., Abrahams, M.R., et al. (2012). Evolution of an HIV glycan-dependent broadly neutralizing antibody epitope through immune escape. Nat. Med. *18*, 1688–1692.

Pancera, M., Shahzad-Ul-Hussan, S., Doria-Rose, N.A., McLellan, J.S., Bailer, R.T., Dai, K., Loesgen, S., Louder, M.K., Staupe, R.P., Yang, Y., et al. (2013). Structural basis for diverse N-glycan recognition by HIV-1-neutralizing V1-V2-directed antibody PG16. Nat. Struct. Mol. Biol. *20*, 804–813.

Pegu, A., Yang, Z.Y., Boyington, J.C., Wu, L., Ko, S.Y., Schmidt, S.D., McKee, K., Kong, W.P., Shi, W., Chen, X., et al. (2014). Neutralizing antibodies to HIV-1 envelope protect more effectively in vivo than those to the CD4 receptor. Sci. Transl. Med. *6*, 243ra88.

Pritchard, L.K., Spencer, D.I., Royle, L., Bonomelli, C., Seabright, G.E., Behrens, A.J., Kulp, D.W., Menis, S., Krumm, S.A., Dunlop, D.C., et al. (2015). Glycan clustering stabilizes the mannose patch of HIV-1 and preserves vulnerability to broadly neutralizing antibodies. Nat. Commun. *6*, 7479.

Pugach, P., Ozorowski, G., Cupo, A., Ringe, R., Yasmeen, A., de Val, N., Derking, R., Kim, H.J., Korzun, J., Golabek, M., et al. (2015). A native-like SOSIP.664 trimer based on an HIV-1 subtype B env gene. J. Virol. *89*, 3380–3395.

Rappuoli, R., Bottomley, M.J., D'Oro, U., Finco, O., and De Gregorio, E. (2016). Reverse vaccinology 2.0: Human immunology instructs vaccine antigen design. J. Exp. Med. *213*, 469–481.

Sanders, R.W., Derking, R., Cupo, A., Julien, J.P., Yasmeen, A., de Val, N., Kim, H.J., Blattner, C., de la Peña, A.T., Korzun, J., et al. (2013). A next-generation cleaved, soluble HIV-1 Env trimer, BG505 SOSIP.664 gp140, expresses multiple epitopes for broadly neutralizing but not non-neutralizing antibodies. PLoS Pathog. *9*, e1003618.

Sanders, R.W., van Gils, M.J., Derking, R., Sok, D., Ketas, T.J., Burger, J.A., Ozorowski, G., Cupo, A., Simonich, C., Goo, L., et al. (2015). HIV-1 VACCINES. HIV-1 neutralizing antibodies induced by native-like envelope trimers. Science *349*, aac4223.

Seaman, M.S., Janes, H., Hawkins, N., Grandpre, L.E., Devoy, C., Giri, A., Coffey, R.T., Harris, L., Wood, B., Daniels, M.G., et al. (2010). Tiered categorization of a diverse panel of HIV-1 Env pseudoviruses for assessment of neutralizing antibodies. J. Virol. *84*, 1439–1452.

Shingai, M., Donau, O.K., Plishka, R.J., Buckler-White, A., Mascola, J.R., Nabel, G.J., Nason, M.C., Montefiori, D., Moldt, B., Poignard, P., et al. (2014). Passive transfer of modest titers of potent and broadly neutralizing anti-HIV monoclonal antibodies block SHIV infection in macaques. J. Exp. Med. *211*, 2061–2074.

Sok, D., Laserson, U., Laserson, J., Liu, Y., Vigneault, F., Julien, J.P., Briney, B., Ramos, A., Saye, K.F., Le, K., et al. (2013). The effects of somatic hypermutation on neutralization and binding in the PGT121 family of broadly neutralizing HIV antibodies. PLoS Pathog. *9*, e1003754.

Sok, D., Doores, K.J., Briney, B., Le, K.M., Saye-Francisco, K.L., Ramos, A., Kulp, D.W., Julien, J.P., Menis, S., Wickramasinghe, L., et al. (2014). Promiscuous glycan site recognition by antibodies to the high-mannose patch of gp120 broadens neutralization of HIV. Sci. Transl. Med. *6*, 236ra63.

Stewart-Jones, G.B., Soto, C., Lemmin, T., Chuang, G.Y., Druz, A., Kong, R., Thomas, P.V., Wagh, K., Zhou, T., Behrens, A.J., et al. (2016). Trimeric HIV-1-Env structures define glycan shields from clades A, B, and G. Cell *165*, 813–826.

Walker, L.M., Phogat, S.K., Chan-Hui, P.Y., Wagner, D., Phung, P., Goss, J.L., Wrin, T., Simek, M.D., Fling, S., Mitcham, J.L., et al.; Protocol G Principal Investigators (2009). Broad and potent neutralizing antibodies from an African donor reveal a new HIV-1 vaccine target. Science *326*, 285–289.

Walker, L.M., Simek, M.D., Priddy, F., Gach, J.S., Wagner, D., Zwick, M.B., Phogat, S.K., Poignard, P., and Burton, D.R. (2010). A limited number of antibody specificities mediate broad and potent serum neutralization in selected HIV-1 infected individuals. PLoS Pathog. *6*, e1001028.

Walker, L.M., Huber, M., Doores, K.J., Falkowska, E., Pejchal, R., Julien, J.P., Wang, S.K., Ramos, A., Chan-Hui, P.Y., Moyle, M., et al.; Protocol G Principal Investigators (2011). Broad neutralization coverage of HIV by multiple highly potent antibodies. Nature *477*, 466–470.

Ward, A.B., and Wilson, I.A. (2017). The HIV-1 envelope glycoprotein structure: nailing down a moving target. Immunol. Rev. *275*, 21–32.

West, A.P., Jr., Scharf, L., Scheid, J.F., Klein, F., Bjorkman, P.J., and Nussenzweig, M.C. (2014). Structural insights on the role of antibodies in HIV-1 vaccine and therapy. Cell *156*, 633–648.

Xiao, X., Chen, W., Feng, Y., Zhu, Z., Prabakaran, P., Wang, Y., Zhang, M.Y., Longo, N.S., and Dimitrov, D.S. (2009). Germline-like predecessors of broadly neutralizing antibodies lack measurable binding to HIV-1 envelope glycoproteins: implications for evasion of immune responses and design of vaccine immunogens. Biochem. Biophys. Res. Commun. *390*, 404–409.

Zolla-Pazner, S., Powell, R., Yahyaei, S., Williams, C., Jiang, X., Li, W., Lu, S., Wang, S., Upadhyay, C., Hioe, C.E., et al. (2016). Rationally designed vaccines targeting the V2 region of HIV-1 gp120 induce a focused, cross-clade-reactive, biologically functional antibody response. J. Virol. *90*, 10993–11006.

Cell Reports, Volume *21*

Supplemental Information

Elicitation of Neutralizing Antibodies

Targeting the V2 Apex of the HIV Envelope

Trimer in a Wild-Type Animal Model

James E. Voss, Raiees Andrabi, Laura E. McCoy, Natalia de Val, Roberta P. Fuller, Terrence Messmer, Ching-Yao Su, Devin Sok, Salar N. Khan, Fernando Garces, Laura K. Pritchard, Richard T. Wyatt, Andrew B. Ward, Max Crispin, Ian A. Wilson, and Dennis R. Burton

A

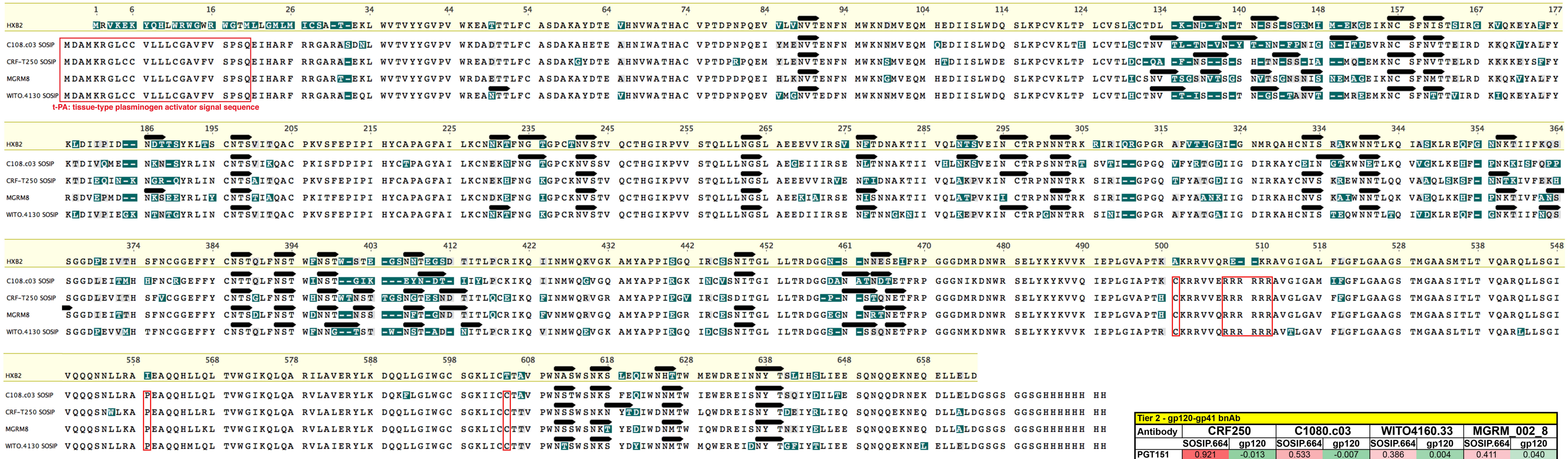

B

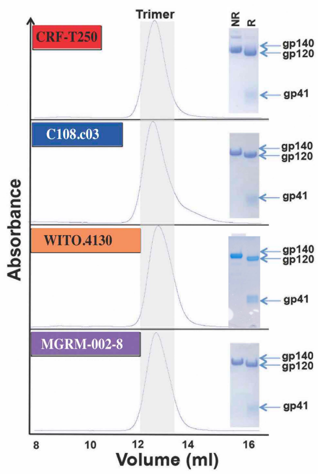

C

Tier 2 - V2 Apex bnAb								
Antibody	CRF250		C1080.c03		WITO4160.33		MGRM_002_8	
	SOSIP.664	gp120	SOSIP.664	gp120	SOSIP.664	gp120	SOSIP.664	gp120
PG9	0.605	0.047	0.480	0.175	0.428	0.067	0.218	0.173
PG16	0.639	0.039	0.531	0.108	0.360	0.055	0.287	0.113
PGT141	0.664	0.021	0.368	0.002	0.680	0.020	0.487	0.057
PGT142	0.787	0.022	0.369	-0.010	0.791	0.008	0.515	0.053
PGT143	0.710	0.022	0.385	-0.002	0.773	0.000	0.343	0.059
PGT144	0.581	0.025	0.192	-0.005	0.667	0.005	0.070	0.078
PGT145	0.878	-0.002	0.614	0.001	0.834	0.014	0.469	0.034
PGDM1400	0.817	-0.007	0.798	0.003	0.598	0.012	0.452	0.052
CH01	0.324	-0.013	0.454	0.008	0.255	0.014	0.146	0.079
CH02	0.232	-0.014	0.301	-0.008	0.291	0.006	0.091	0.039
CH03	0.347	-0.005	0.401	-0.004	0.233	0.002	0.099	0.062
CH04	0.244	-0.013	0.351	0.001	0.188	0.008	0.123	0.079
CAP256.01	0.634	0.041	0.160	0.030	0.059	0.061	0.268	0.076
CAP256.02	0.779	0.018	0.206	0.008	0.089	0.034	0.399	0.066
CAP256.03	0.821	0.031	0.383	0.038	0.065	0.052	0.475	0.063
CAP256.04	0.746	0.031	0.282	0.036	0.071	0.049	0.413	0.063
CAP256.05	0.856	0.025	0.161	0.027	0.067	0.034	0.346	0.061
CAP256.06	0.507	0.014	0.037	0.004	0.663	0.016	0.247	0.047
CAP256.07	0.476	-0.003	0.025	-0.002	0.043	0.010	0.177	0.039
CAP256.08	0.676	0.014	0.430	0.029	0.048	0.040	0.431	0.097
CAP256.09	0.725	0.015	0.416	0.038	0.044	0.037	0.493	0.073
CAP256.10	0.566	0.018	0.165	0.028	0.096	0.040	0.329	0.054
CAP256.11	0.627	0.039	0.082	0.036	0.075	0.043	0.323	0.076
CAP256.12	0.348	0.022	0.021	0.032	0.054	0.034	0.202	0.072
CAP256.I1	0.509	0.042	0.022	0.031	0.055	0.062	0.178	0.075
CAP256.I2	0.751	0.028	0.120	0.030	0.058	0.054	0.338	0.065
non-neutralizing V2 Apex								
2909	0.066	0.037	0.032	0.039	0.063	0.053	0.062	0.071
C108G	0.078	0.054	0.055	0.068	0.078	0.077	0.069	0.083
830A	0.052	0.020	0.052	0.206	0.177	0.482	0.135	0.135
697	0.069	0.052	0.035	0.233	0.092	0.182	0.109	0.245

Tier 2 - V3-glycan bnAb								
Antibody	CRF250		C1080.c03		WITO4160.33		MGRM_002_8	
	SOSIP.664	gp120	SOSIP.664	gp120	SOSIP.664	gp120	SOSIP.664	gp120
PGT121	0.813	0.166	0.036	0.002	0.316	0.164	0.351	0.073
PGT122	0.691	0.063	0.021	0.015	0.169	0.118	0.236	0.224
PGT123	0.899	0.173	0.037	0.001	0.378	0.215	0.425	0.245
PGT124	0.762	0.240	0.007	0.003	0.236	0.308	0.381	0.431
PGT125	0.651	0.157	0.530	0.197	0.653	0.065	0.486	0.481
PGT126	0.676	0.185	0.545	0.106	0.610	0.081	0.539	0.635
PGT127	0.508	0.048	0.277	-0.003	0.216	0.016	0.335	0.405
PGT128	0.731	0.190	0.478	0.111	0.622	0.052	0.464	0.651
PGT130	0.595	0.156	0.431	0.133	0.361	0.047	0.314	0.542
PGT131	0.661	0.144	0.421	0.084	0.207	0.068	0.275	0.514
PGT133	0.804	0.232	0.024	0.022	0.227	0.325	0.382	-0.001
10-1074	0.823	0.270	0.033	0.021	0.267	0.362	0.415	0.431
PGT135	0.510	0.116	0.006	-0.066	0.304	0.247	0.193	0.119
PGT136	0.067	0.022	0.012	-0.004	0.328	0.199	0.143	0.079
PGT137	0.539	0.131	0.012	-0.016	0.467	0.168	0.193	0.202
2G12	0.559	0.241	0.314	-0.010	0.556	0.229	0.420	0.215

Tier 2 - CD4 binding site bnAb								
Antibody	CRF250		C1080.c03		WITO4160.33		MGRM_002_8	
	SOSIP.664	gp120	SOSIP.664	gp120	SOSIP.664	gp120	SOSIP.664	gp120
VRC01	0.063	0.011	0.050	0.130	0.143	0.114	0.140	0.275
VRC03	0.062	0.010	0.017	0.023	0.253	0.038	0.077	0.104
VRC06	0.023	-0.003	-0.004	0.011	0.037	0.022	0.037	0.064
HJ16	0.044	0.005	0.022	0.023	0.065	0.050	0.058	0.065
12A12	0.113	0.046	0.138	0.231	0.175	0.198	0.166	0.375
NIH45-46	0.409	0.024	0.056	0.112	0.134	0.061	0.188	0.354
PGV04	0.049	0.033	0.012	0.093	0.097	0.088	0.094	0.271
CH103	0.075	0.060	0.029	0.047	0.074	0.076	0.096	0.299
3BNC60	0.108	0.015	0.083	0.144	0.289	0.142	0.204	0.281
3BNC117	0.090	0.009	0.061	0.135	0.290	0.092	0.170	0.253
b6	0.128	0.614	0.041	0.062	0.145	0.354	0.282	0.651
b12	0.057	-0.025	0.281	0.266	0.251	0.299	0.401	0.542
F105	0.037	0.452	-0.019	-0.019	0.075	0.412	0.164	0.514
CD4-IgG2	0.382	0.589	0.382	0.589	0.293	0.565	0.540	0.850

Tier 2 - gp120-gp41 bnAb								
Antibody	CRF250		C1080.c03		WITO4160.33		MGRM_002_8	
	SOSIP.664	gp120	SOSIP.664	gp120	SOSIP.664	gp120	SOSIP.664	gp120
PGT151	0.921	-0.013	0.533	-0.007	0.386	0.004	0.411	0.040
PGT152	0.846	-0.013	0.615	-0.002	0.328	0.001	0.316	0.041
PGT153	0.616	-0.004	0.294	0.012	0.094	0.020	0.088	0.035
PGT154	0.774	0.009	0.192	0.021	0.090	0.040	0.188	0.049
PGT155	0.854	0.022	0.458	0.026	0.244	0.033	0.297	0.058
PGT156	0.865	0.024	0.512	0.034	0.241	0.045	0.369	0.085
PGT157	0.905	0.051	0.384	0.044	0.213	0.078	0.307	0.091
PGT158	0.928	0.033	0.485	0.033	0.203	0.048	0.310	0.078
8ANC195	-0.002	-0.023	0.056	0.152	0.163	-0.016	0.214	-0.001
*35022	0.373	-0.058	0.079	-0.066	0.023	-0.063	0.122	-0.011

Non-neutralizing V3								
Antibody	CRF250		C1080.c03		WITO4160.33		MGRM_002_8	
	SOSIP.664	gp120	SOSIP.664	gp120	SOSIP.664	gp120	SOSIP.664	gp120
447-52D	0.207	0.402	0.042	0.028	0.585	0.591	0.413	0.405
2557	0.356	0.608	0.112	0.157	0.367	0.321	0.354	0.640
4022	0.385	0.637	0.085	0.161	0.323	0.331	0.391	0.639
3074	0.290	0.682	0.242	0.497	0.317	0.467	0.362	0.693
3791	0.333	0.733	0.124	0.275	0.074	0.065	0.350	0.666
3904	0.144	0.677	0.098	0.433	0.075	0.054	0.124	0.598
4508	0.057	0.027	0.012	0.026	0.064	0.036	0.063	0.073
537	0.038	0.022	0.017	0.035	0.090	0.215	0.050	0.060
15e	0.057	0.088	0.104	0.380	0.110	0.290	0.091	0.224
19b	0.268	0.480	0.331	0.453	0.533	0.420	0.336	0.481
14e	0.291	0.573	0.415	0.564	0.472	0.508	0.442	0.635

Non-neutralizing CD4i								
Antibody	CRF250		C1080.c03		WITO4160.33		MGRM_002_8	
	SOSIP.664	gp120	SOSIP.664	gp120	SOSIP.664	gp120	SOSIP.664	gp120
A32	0.085	0.510	0.080	0.609	0.126	0.552	0.087	0.647
17b	0.059	0.050	0.009	0.078	0.099	0.348	0.418	0.157
E51	0.058	0.023	0.034	0.065	0.145	0.284	0.465	0.102

Non-neutralizing gp41								
Antibody	CRF250		C1080.c03		WITO4160.33		MGRM_002_8	
	SOSIP.664	gp120	SOSIP.664	gp120	SOSIP.664	gp120	SOSIP.664	gp120
F240	0.007	-0.008	0.001	-0.004	0.014	-0.006	0.005	0.006

negative control								
Antibody	CRF250		C1080.c03		WITO4160.33		MGRM_002_8	
	SOSIP.664	gp120	SOSIP.664	gp120	SOSIP.664	gp120	SOSIP.664	gp120
DEN3	-0.005	-0.023	-0.028	-0.016	-0.002	-0.027	-0.012	-0.001

Figure S1. Immunogen sequences, purification, and antibody reactivities, Related to Figures 1 and 2. A) Immunogen sequences C108.c03 (AE) (JN944660.1), CRF T-250 (AG) (EU513189.1), MGRM-002-8 (AG) (MF510462) and WITO.4130 (B) (AY835451.1) gp140 ectodomain regions (20-664 HXB2 numbering), were adapted to the SOSIP format by replacing the REKR furin cleavage site with R6 (RRRRRR), and by making the following mutations: A501C, I559P, T605C (red rectangles). The tissue-type plasminogen activator signal sequence was used and constructs with or without a C-terminal 8x His were made. The sequence was cloned into the multiple cloning site of the phCMV expression vector under control of the ubiquitously active CMV promoter. **B)** Size exclusion chromatography (SEC) elution volumes (280nm absorbance) for PGT145 purified SOSIP trimers and their corresponding SDS-PAGE reducing and non-reducing gels. S200 10/30 columns were equilibrated with PBS before injecting PGT145 eluted SOSIP from a 500ul injection loop. Trimer fractions were pooled, aliquoted, and frozen at -80°C. **C)** SOSIP trimers or sequence-matched gp120 proteins binding to mAbs are arranged by epitope. Values are given as response units (RUs) obtained using Biolayer Interferometry.10ug/ml of mAb is bound to anti-human IgG Fc sensors and maximum binding after 60s is recorded in 300nM SOSIP or 500nM gp120. Color scale is red to green from higher to lower binding.

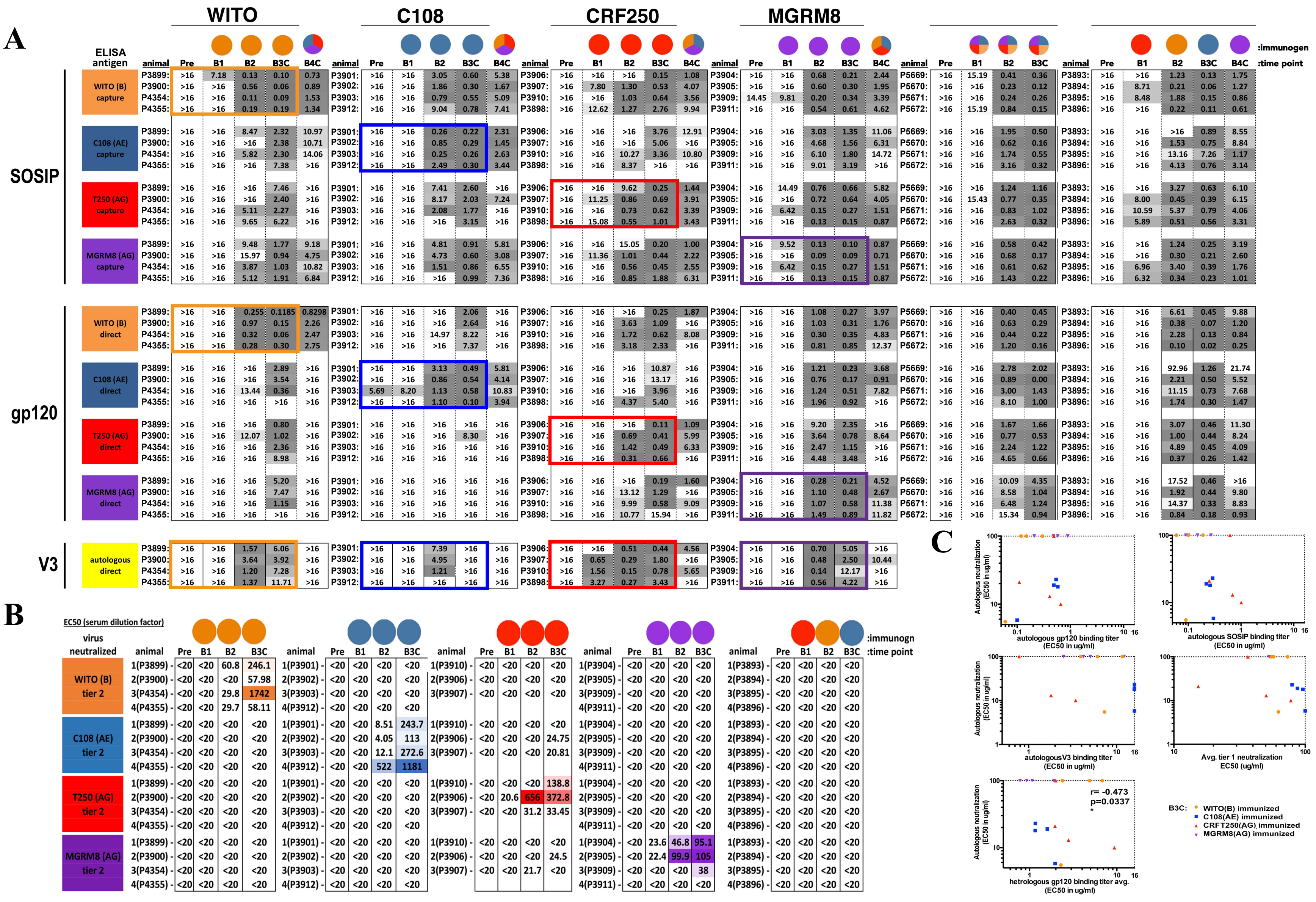

Figure S2. Binding titers, serum neutralization and data correlations for trimer-immunized rabbits, Relating to Figure 3. A) ELISA was used to measure binding titers to autologous and heterologous SOSIPs, gp120 and autologous V3 peptides. The table is organized with ELISA antigens given in the left column as colored boxes. Sample collection points for the different immunization groups (WITO: orange, C108: blue, CRF250: red, MGRM8: purple) are indicated across the top. Pre, B1, B2 and B3C correspond to the time points depicted in Figure 3A. Rabbit ID is given to the left of each immunization group. 50% maximum binding EC_{50}s in µg/ml of rabbit immune serum IgG are given. A darker grey colored box indicates higher binding titers. **B)** Autologous neutralization midpoint concentrations (IC_{50}s) as serum dilution factors calculated as was done for serum purified IgG in Figure 3B, for rabbits immunized with either WITO, C108, CRF250, MGRM8 or sequentially. The pseudovirus neutralized is on the far left. The rabbit ID is to the left of IC_{50}s organized by group across the page. Each sample time point is indicated at the top of the row. **C)** Autologous neutralization (IC_{50} in µg/ml rabbit immune serum IgG) at the B3C time point (when maximum autologous neutralization was observed) for the four groups immunized with individual SOSIPs were plotted against SOSIP, gp120 or V3 binding titers to identify possible correlates for the development of autologous neutralizing responses. Data points are colored according to the immunogen given. Heterologous binding titers are an average of the three heterologous titers.

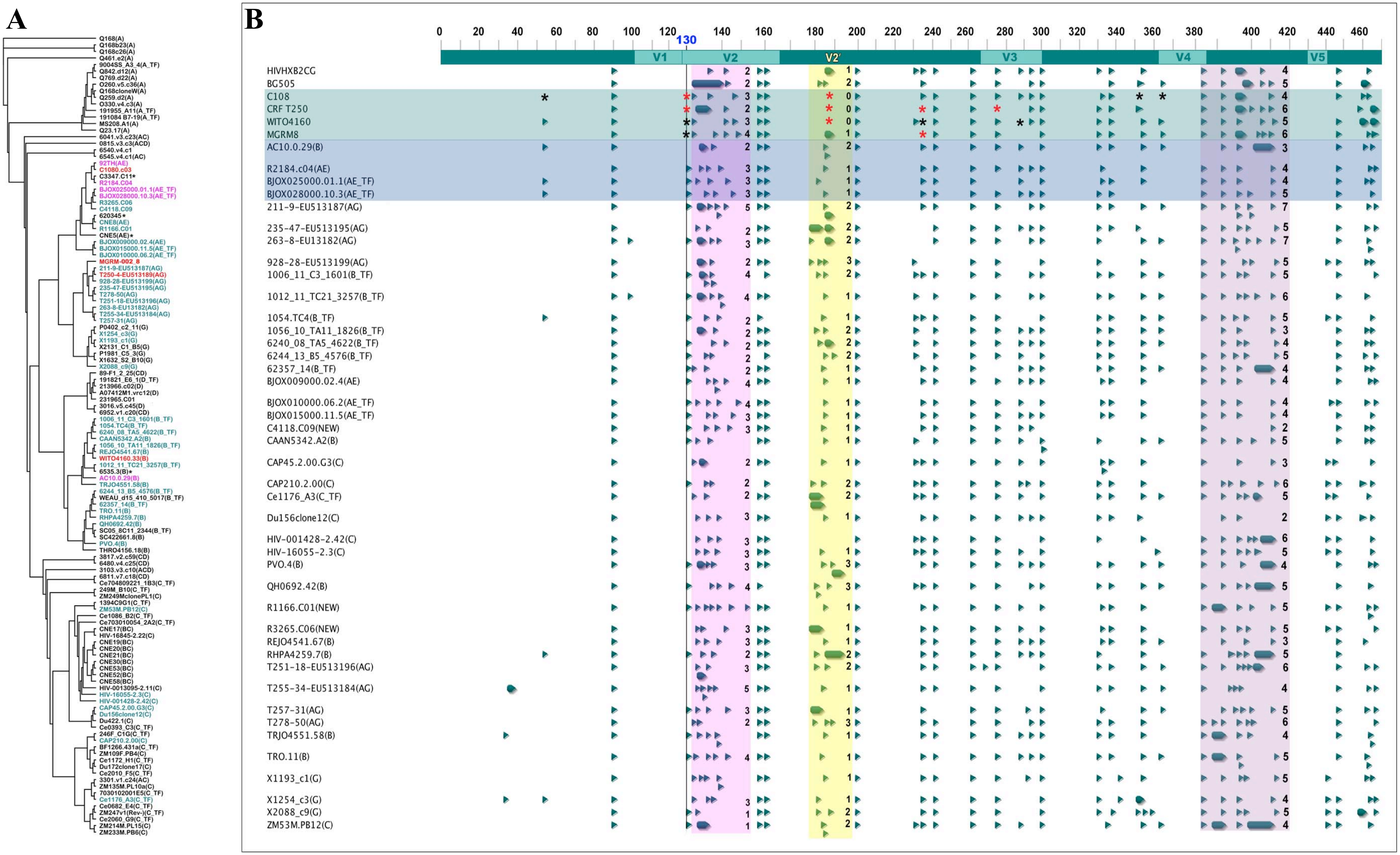

Figure S3. Phylogenic tree and glycan alignment of select tier 2 viruses. Relating to Figures 3-5. A) Phylogenic relationships of Tier 2 viruses screened for neutralizing breadth by rabbit immune serum IgG. Viruses screened were selected based on their similarity to immunogens by alignment (entire Env or V2 region) (colored). Immunogens sequences are in red. Heterologous viruses neutralized by rabbit P5672 are in pink. Viruses labeled with asterisks had poor infectivity and could not be used in neutralization assays. Viruses in cyan were tested but could not be neutralized. Four of the heterologous viruses neutralized are clade AE and cluster with C108, while one clade B (AC10) is most similar to the WITO immunogen, which failed to induce autologous neutralizing titers in this animal (P5672). **B)** Glycan alignment of gp120s from viruses that were screened for neutralizing breadth with rabbit immune serum IgG. Row at the top indicates region of the protein with numbering according to the HXB2 reference. Cyan arrows are NXT/S sites. Vertical columns of colored blocks have glycan sites within this sequence stretch summed to the right of the colored block. Black asterisk represent glycan hole sites that were filled and had no effect on rabbit immune serum IgG neutralization. Red asterisks indicate glycan hole sites that, when filled by adding an NXS/T site, attenuate/eliminate rabbit immune serum IgG neutralization (Figure 5).

A

B

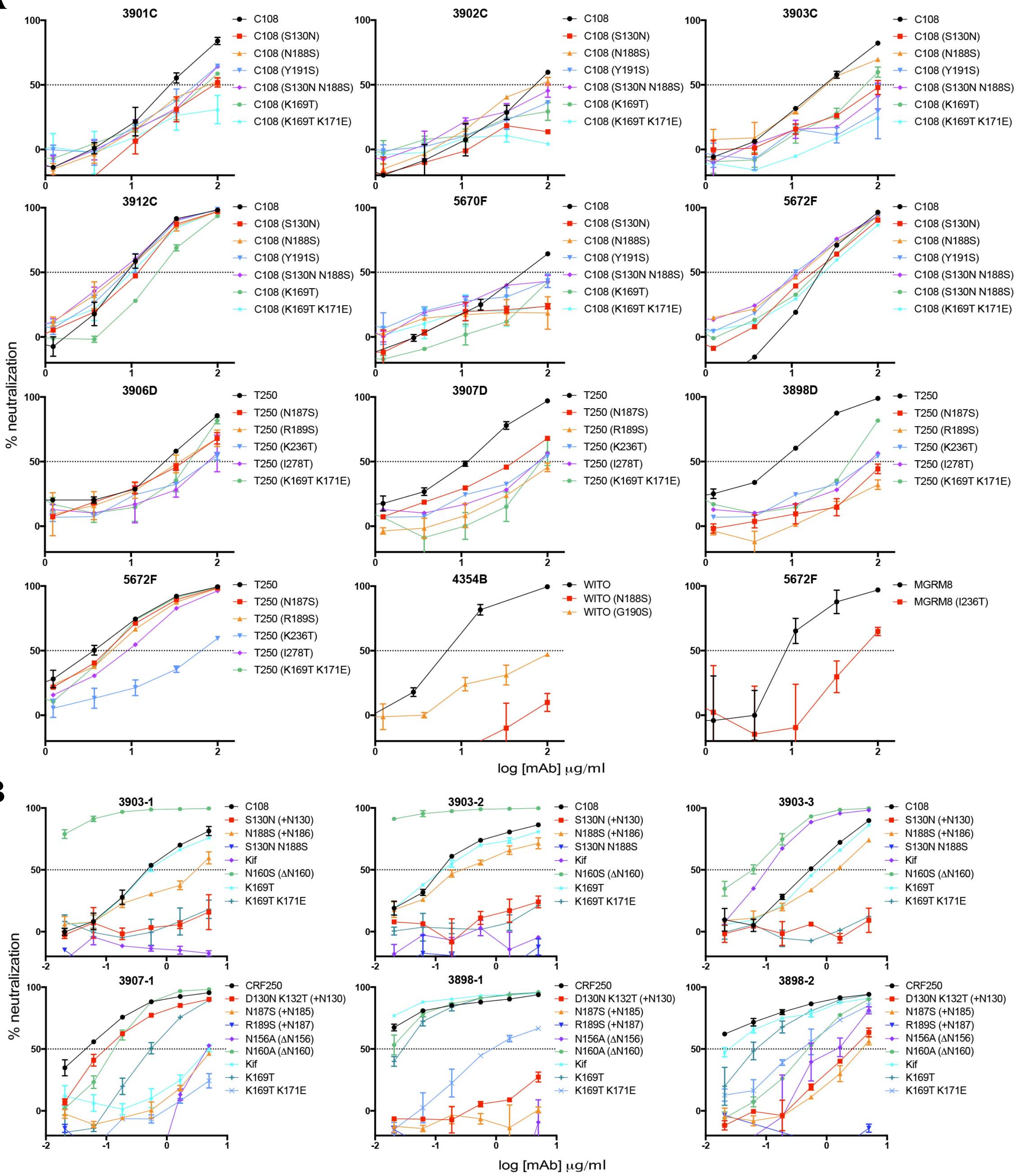

Figure S4. Neutralization titration curves using select virus mutants, Related to Figures 4 and 5. A) % neutralization is given as a function of the log of the polyclonal IgG concentration (in μg/ml) that was purified from immune serum at time B3 from the animal indicated in the title of the graph. IC_{50}s in Figure 4 are derived from these curves. **B)** % neutralization is given as a function of the mAb concentration in μg/ml for the mAbs indicated in the title of the graph. IC_{50}s in Figure 5 are derived from these curves. Viruses neutralized are given in the legends to the right of each graph. Virus mutations add PNG sites at 130, the V2' region, 234 or 276, or remove PNG sites at N156 or N160. Strand C mutants were also tested. Reduction or elimination of neutralization by select mutants helps to map neutralizing responses to specific epitopes on the wild type virus. Error bars represent SD between replicate data points.

A

B

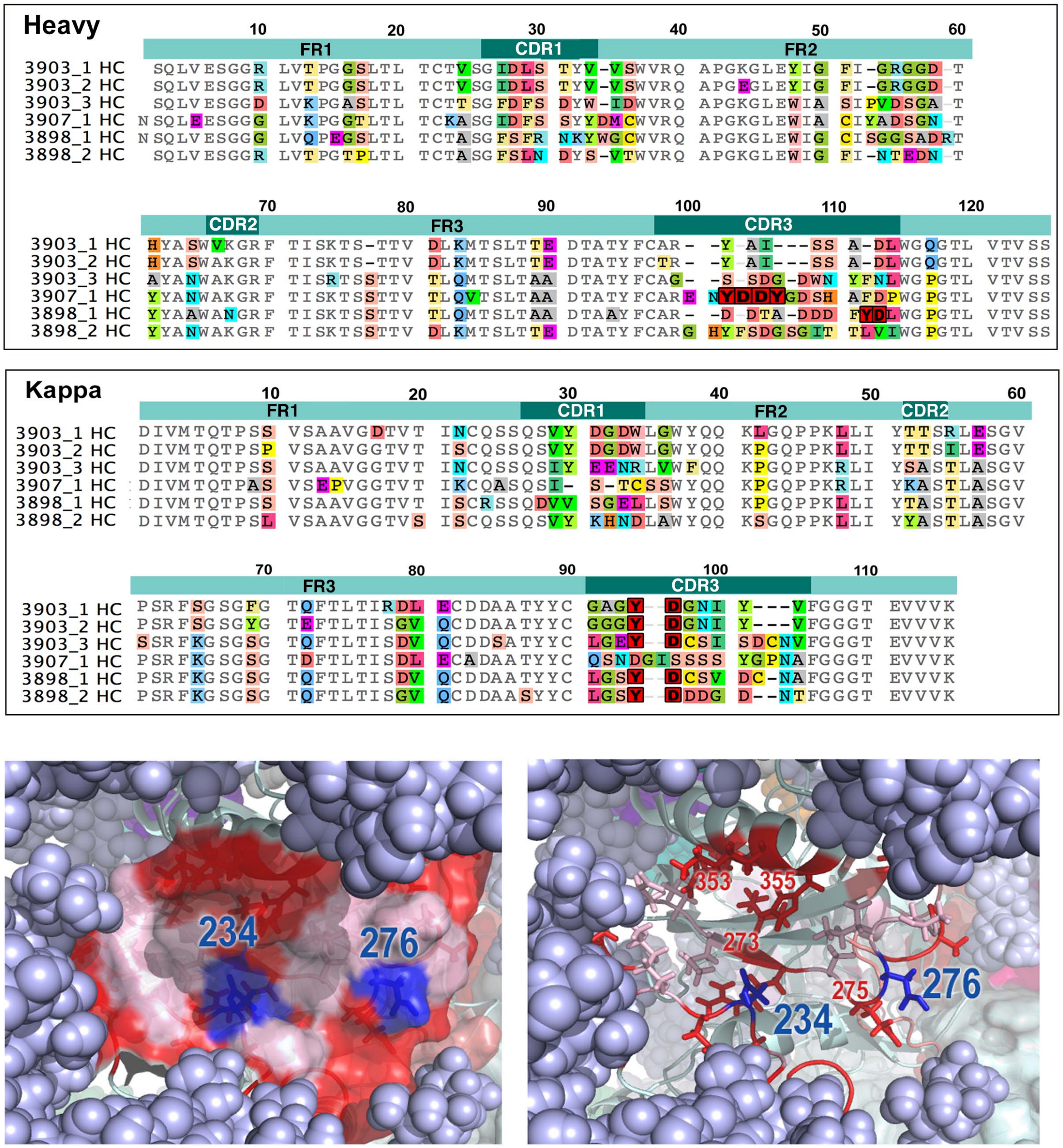

Figure S5. Alignment of rabbit neutralizing mAb sequences and depiction of an alternate glycan hole present in CRF250 and MGRM8 immunogens. Related to Figures 4 and 5. A) Heavy chain and kappa light chain VDJ and VJ genes respectively. Linear diagram above indicates framework and complementarity determining regions. Numbering is based on the consensus from all sequences. Positions at which sequences differ are colored by amino acid. YD motifs in CDR3s are represented in highlighted red blocks. **B)** A glycan hole proximal to the CD4 binding site and centered on glycans absent at 234 (CRF250/MGRM8), and 276 (CRF250). BG505 SOSIP trimer (PDB 4TVP) with glycans modeled in (also used in McCoy, LE et al. 2016) was used as a model to show a surface rendered image (on the left) or residue backbones and side chains (on the right), centered on the 234/276 site. Conserved residues between CRF250 and MGRM8 are shown in red, while residues that differ between them are in light pink. Surrounding modeled glycans are shown as violet and the location of this region in the context of the trimer and its position relative to the CD4 binding site is shown in Figure 4D.
